# Is Short-Read 16S rRNA Sequencing of Oral Microbiome Sampling a Suitable Diagnostic Tool for Head and Neck Cancer?

**DOI:** 10.3390/pathogens13100826

**Published:** 2024-09-24

**Authors:** Kenny Yeo, Fangmeinuo Wu, Runhao Li, Eric Smith, Peter-John Wormald, Rowan Valentine, Alkis James Psaltis, Sarah Vreugde, Kevin Fenix

**Affiliations:** 1 Discipline of Surgery, Adelaide Medical School, The University of Adelaide, Adelaide, SA 5000, Australia; fangmeinuo.wu@adelaide.edu.au (F.W.); runhao.li@adelaide.edu.au (R.L.); eric.smith@adelaide.edu.au (E.S.); alkis.psaltis@adelaide.edu.au (A.J.P.); sarah.vreugde@adelaide.edu.au (S.V.); 2 Department of Surgery-Otolaryngology Head and Neck Surgery, The University of Adelaide and the Basil Hetzel Institute for Translational Health Research, Central Adelaide Local Health Network, Woodville South, SA 5011, Australia; rowan.valentine@sa.gov.au; 3 Department of Haematology and Oncology, Basil Hetzel Institute for Translational Health Research and the Queen Elizabeth Hospital, Central Adelaide Local Health Network, Woodville South, SA 5011, Australia; 4 Department of Surgery-Otolaryngology Head and Neck Surgery, The University of Adelaide, Adelaide, SA 5000, Australia; peterj.wormald@adelaide.edu.au

**Keywords:** oral microbiome, 16S ribosomal RNA, saliva, oral rinse, head and neck cancer

## Abstract

The oral microbiome, studied by sampling the saliva or by oral rinse, has been long thought to have diagnostic capacity for head and neck cancers (HNC). However, previous reports on the HNC oral microbiome provide inconsistent results. The aim of this study is to consolidate these datasets and determine the oral microbial composition between HNC patients to healthy and premalignant individuals. We analyzed 16 published head and neck cancer (HNC) short-read 16S rRNA sequencing datasets, specifically targeting the V3V4, V4 and V4V5 regions. These datasets included saliva and oral rinse samples from donors with HNC, as well as from healthy and premalignant donors. Differences in diversities and microbial abundance were determined. HNC saliva displayed lower alpha diversity than healthy donors. In contrast, the opposite trend was observed for oral rinse samples. Beta diversity scores were largely similar across different patient types. Similar oral phyla were detected for all samples, but proportions were largely dependent on sample type (i.e., saliva or oral rinse) and primer set utilized for 16S rRNA sequencing. *Neisseria*, *Leptotrichia* and *Megasphaera* were elevated in healthy saliva, while *Mycoplasma* was elevated in HNC saliva. Oral rinse and saliva displayed similar enrichment for *Fusobacterium*, while *Veillonella*, *Alloprevotella*, and *Campylobacter* showed conflicting results. The sparse partial least squares discriminant analysis model performed effectively in discriminating HNC from healthy or premalignant patients using V3V4 saliva (AUC = 0.888) and V3V4 oral rinse (AUC = 0.928), while poor discriminative capacity was observed for V4 saliva (AUC = 0.688). In conclusion, our meta-analysis highlighted the limitations of 16S rRNA sequencing, particularly due to variations across study batches, primer sets (i.e., V3V4, V4), and sample types. Hence, caution should be exercised when interpreting 16S rRNA sequencing results across studies, especially when different primer sets and sample types are used.

## 1. Introduction

Oral microbes have been associated with cancer and other diseases such as inflammatory bowel disease, periodontitis, and cardiovascular disease [1]. Oral microbes have been identified within cancer tissues [2], and the oral sampling of cancer patients suggests the presence of a dysregulated oral microbiome [3]. Thus, the oral microbiome is viewed as a possible non-invasive, safe, and accessible diagnostic tool for cancer diagnostics. Head and neck cancer (HNC) is a multi-faceted disease, with approximately 90% of these cases derived from distinct mucosal epithelial regions (termed as squamous cell carcinomas) such as the oral cavity, oropharyngeal, laryngeal, hypopharyngeal, and nasopharynx [4]. At the early stage (T1–T2), the disease is usually asymptomatic, with physical examination remaining as the best intervention [5,6,7]. However, most patients (>60%) are diagnosed at a later stage (T3–T4), resulting in a reduced treatment window and lower overall survival (OS) rates (5-year OS = 36%) [5]. This situation is worse in lower-income countries, where late-stage disease has a 5-year OS of only 3% [5]. Hence, it is crucial to have an affordable, rapid, user-friendly and minimally invasive diagnostic tool for HNC detection screening.

The 16S ribosomal RNA (rRNA) sequencing of oral samplings has been utilized in HNC studies to study differences in the microbiome between cancer, premalignant and healthy oral samples [8]. Oral samples are primarily collected from saliva [9,10,11,12,13,14,15,16,17,18,19,20,21,22,23,24,25,26], oral rinse [27,28,29,30], or by swabbing oral sites [8]. Collecting saliva or oral rinse is straightforward and involves the participant providing a sample into a tube. Oral rinse involves using a fixed volume of solution (~10 mL), like normal saline [28,30,31,32,33,34] or distilled water [35]. These methods attempt to sample the oral cavity without sampling a particular site, making them more practical for diagnostic screening. In contrast, swabbing involves collecting samples from specific sites, such as buccal or gingival areas, the tongue, or tumour-adjacent areas, as seen in previous HNC oral microbiome studies (reviewed in meta-analyses [8,36,37,38]). Since oral microbes are site-specific, with some bacteria preferring particular locations [39], data from swabs can be biased based on the collection site, making diagnostics dependent on region-specific sampling [24,40]. Therefore, saliva or oral rinse may be more suitable, and uniform methods for developing oral microbiome diagnostics compared to swabs.

The healthy core salivary microbiome primarily consists of the bacterial phyla *Firmicutes*, *Proteobacteria*, *Actinobacteria*, *Bacteroidetes*, and *Fusobacteria*, with the most common genera being *Streptococcus*, *Prevotella*, and *Neisseria* [41]. Importantly, numerous host factors such as age, diet, gender, smoking, alcohol consumption, disease status (e.g., periodontitis, cancer) and treatment have been shown to affect microbial composition [41]. Alpha and beta diversities are commonly compared between saliva of HNC and healthy individuals; however, conflicting results prevent the formation of a consensus microbial diversity shift between HNC and healthy states [8,16,17,24,29]. As an example, *Fusobacterium* is inconsistently reported as being elevated in either HNC [29,42] or healthy [10,16,24] saliva samples. We and others have shown that elevated *Fusobacterium* in HNC tissues is associated with better overall survival in HNC [27,43,44,45]. However, it is unclear if the abundance of *Fusobacterium* in the saliva can be linked to disease status. Moreover, inconsistencies were also reported for other known beneficial (*Bifidobacterium*) or oral commensal (*Streptococcus* and *Prevotella*) bacterial genera [9,10,14,24,29]. Oral rinse sampling also displayed some inconsistencies in reporting for alpha and beta diversities between HNC and healthy samples [27,28,30,31,32,33,34,35,46,47]. However, unlike saliva sampling, taxa-level information related to oral rinse was more consistent across studies [27,28,30,31,32,33,34,35,46,47].

Other than microbiome profiling, studies have also evaluated the diagnostic potential (i.e., in discriminating cancer from healthy) of using specific microbes detected in the saliva [12,13,20,21,48,49,50] or oral rinse [27,30,31,32,33,34] for HNC. For example, a combination of 12 genera (*Fusobacterium*, *Neisseria*, *Streptococcus*, *Rothia*, *Granulicatella*, *Actinomyces*, *Lautropia*, *Corynebacterium*, *Oribacterium*, *Peptostreptococcus*, *Cardiobacterium* and *Abiotrophia*) has been used to distinguish healthy from HNC oral rinse [27,30]. The reported receiver operating characteristic (ROC) analysis using the 16S rRNA abundance of specific HNC-discriminating microbes, either alone or combined with clinical variables, achieved an area under the ROC curve (AUROC) ranging from 0.63 to 0.95 for saliva samples [12,13,20,21,48,49,50] and 0.62 to 0.98 for oral rinse samples [27,30,31,32,33,34] in comparative studies of HNC and healthy controls/donors. These findings suggest that oral sampling using saliva and oral rinse could potentially serve as a diagnostic tool for HNC. Thus, performing a meta-analysis of published 16S rRNA datasets derived from saliva or oral rinse samplings is necessary to determine if these oral microbiome signatures can be used for HNC diagnostics.

Several systematic reviews and meta-analyses have attempted to provide a consensus HNC oral microbiome signature by using processed [8,36,37,51,52,53,54] or re-analyzed [55] studies of 16S rRNA sequencing data collected from saliva, swab, oral rinse and tissues. Critically, these systematic reviews and meta-analyses have relied on extracting the reported post-analysis microbiome data rather than re-analyses of raw datasets. This neglects important confounders, including data pre-processing, sequencing depth [56,57], inter-patient variations [41], amplification primer biasness [58], and sequencing techniques utilized in individual studies. Additionally, most meta-analyses studies consolidated different oral sampling types as one cohort, even though different oral samplings can exhibit distinct microbial communities [24,39,40,59]. Re-analyses of 16S rRNA oral microbiome data comparing healthy to HNC samples while ignoring the oral sampling utilized [55] may lead to issues with the overall interpretation of results. Hence, in this study, we analyzed how different sampling types (saliva, oral rinse) can affect the HNC oral microbiome. Due to the diverse sampling sites reported with swab collection [8,36,37,38], we chose to focus on saliva and oral rinse sampling. The goal of this meta-analysis was to identify if there were differences in 16S rRNA microbial diversity and taxa-level abundance between HNC, premalignant and healthy individuals in multiple cohorts of saliva and oral rinse samples, while identifying potential confounders that may influence analyses. Moreover, we investigated whether these differences were consistent across saliva and oral rinse sampling methods. Lastly, we explored the potential use of the 16S rRNA sequencing of saliva and oral rinse as a diagnostic tool for HNC.

## 2. Materials and Methods

This study was performed according to the Preferred Reporting Items for Systematic Reviews and Meta-Analyses (PRISMA) Statement (File S1) [60].

### 2.1. Selection Criteria, Database Search and Study Design

The inclusion criteria for this study were: (1) presence of metadata to distinguish sample and subject types; (2) Illumina short-read amplicon 16S rRNA sequencing; (3) saliva or oral rinse. The exclusion criteria for this study were: (1) samples without metadata; (2) other non-Illumina short-read 16S rRNA sequencing technologies; (3) any other sample types that were not saliva and oral rinse; (4) any samples with treatment and/or antibiotic usage. Database searches and extractions were performed on 1 February 2024 by three independent reviewers, and datasets published after this date were not included (Table S1). Risk of bias (RoB) was assessed using RoB2 assessment spreadsheet (ROB2_IRPG_beta_v9) and search terms are provided in Table S1 [61].

### 2.2. Downloading, Pre-Processing and Metadata Collection of 16S rRNA Datasets

All published raw 16S rRNA sequencing datasets were downloaded from NCBI SRA using SRA Toolkit [62]. Metadata for all samples were obtained from NCBI SRA or directly defined based on information provided in the publications. Primers and adapters were removed using cutadapt plugin in QIIME2 v2024.5, followed by denoising using QIIME2 plugin, Divisive Amplicon Denoising Algorithm 2 (DADA2) [63,64,65]. Sequences from different studies were then merged into a table before classification using QIIME2 and SILVA reference database (version silva-138-99-nb-classifier) [64]. The methods of pre-processing raw microbial data have previously been described [43]. Briefly, bacteria amplicon sequence variants (ASVs) were agglomerated into the genus level and low abundances of ASVs were filtered using PreFL from PLSDA-batch v1 (keep.spl = 10, keep.var = 0.01), while samples with less than 1000 reads were also removed using phyloseq v1.48 [66,67]. The total remaining samples per study is detailed in Table S2. Overall, this study included 1423 samples collected using saliva (HNC = 508, healthy = 407, premalignant = 172) or oral rinse (HNC = 206, healthy = 130), as shown in Table S2.

### 2.3. Evaluation and Correcting for Batch Effects

To assess for batch effects and effectiveness after batch correction, we used principal coordinate analysis (PCoA) density plots of Bray–Curtis distance and Permutational Multivariate Analysis of Variance (PERMANOVA) on both adjusted and unadjusted rarefied relative abundance [68]. For compositional data analysis (CoDA), the data were normalized using the central log ratio (CLR), and Euclidean distance of CLR-abundance was used for PCoA. Additional assessments included heatmap clustering analysis, alignment score, partial redundancy analysis (pRDA), and R^2^ assessment, as described previously [43,66].

Batch adjustment was performed using MMUPHin v1.18 (Meta-Analysis Methods with a Uniform Pipeline for Heterogeneity in microbiome studies) in R to adjust for BioProject [68]. MMUPHin was performed only on V3V4 and V4 saliva, as other datasets did not require batch adjustment (File S2). The methods of batch adjustment and evaluation are fully described in File S2. Subsequent analyses were performed on each primer set separately (File S2).

### 2.4. Alpha and Beta Diversity Analysis at the Genus Level

Short-read Illumina 16S rRNA sequencing is largely limited to genus-level resolution; therefore, alpha and beta diversities analyses were performed at the genus level [69]. Conventional and compositional methods of analysis were performed for alpha and beta diversities, as described previously [43,56,70,71,72]. Briefly, samples were rarefied using rarefy_even_depth in phyloseq to sample with least depth (read = 1000) [67]. Alpha diversity was measured using Shannon index, via the microeco v1.9.1 R package [73]. Differences in alpha diversities between HNC, healthy, and premalignant samples within studies were tested using Kruskal–Wallis test with Dunn’s multiple comparison and the Mann–Whitney test.

For beta diversity analysis, the rarefied relative abundances of all genera were ordinated using Bray–Curtis distance and visualized with a PCoA using phyloseq v1.46 and ggpubr v0.6 R packages [67]. PERMANOVA was used to determine differences in beta diversity between HNC, healthy, and premalignant groups [74], with dispersion (variances) assessed using the betadisper test from vegan v2.6 [74]. Since PERMANOVA assumes the homogeneity of dispersion among groups, the betadisper test checked for differences in dispersion; significant differences in dispersion could influence PERMANOVA results. All tests were performed with 999 permutations. To address the compositionality in the microbiome data, CLR-transformation (offset = 0.5) was used to obtain scale-invariant values, mitigating discrepancies in library sizes [56]. The CLR abundance data were then analyzed using Euclidean distances and plotted with a PCoA [68].

### 2.5. Differential Abundance Analysis between HNC, Healthy and Premalignant Oral Samples

Mean relative abundances at both the phylum and genera level were calculated separately for saliva and oral rinse samples using the phyloseq R package. Further differential abundance analyses at the genus level were performed using the linear regression framework for differential abundance analysis v0.2 (LinDA) with the microeco R package [73,75]. LinDA fits a linear model to CLR-transformed abundance data, correcting for compositional effects and biases [75]. We performed LinDA to compare HNC samples to healthy or premalignant oral samples. Analysis at the phylum level was not performed with LinDA, as it is not recommended with a small number of features [75]. *p*-values were adjusted using Bonferroni correction.

### 2.6. Multivariate Sparse Partial Least Squares Discriminant Analysis (sPLS-DA) Classification to Discriminate Oral Sampling

To test if saliva and oral rinse sampling can be discriminated, we applied sparse partial least squares discriminant analysis (sPLS-DA) using the mixOmics v6.28 R package [76]. For the “tune.splsda” function, we used M-folds = 10 and cross-validation = 100 to determine the optimal number of variables and components. The area under curve of the receiver operating characteristics (AUROC) curve was calculated using mixOmics. The AUC value served as a quantification of the discriminatory potential between sample types. A higher AUC value, closer to 1, signified a test approaching perfection in its ability to distinguish between the samples. Heatmaps were generated to visualize the variables that contribute to sample differentiation.

## 3. Results

### 3.1. Study Characteristics and Dataset

**Figure 1:**
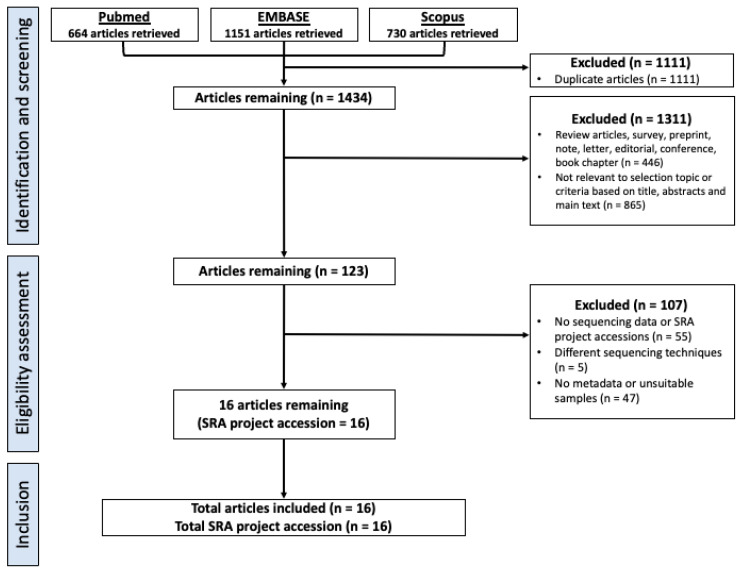
Study selection flowchart.

Our search strategy identified a total of 664 articles from PubMed, 1151 from EMBASE and 730 from Scopus. After removing duplicates, non-articles, and irrelevant articles, 123 articles remained (Figure 1). Following the application of our inclusion criteria, a further 107 articles were excluded, leaving 16 unique project accessions from 16 published articles (Figure 1, Table S2). After pre-processing and filtering, the final dataset included 336 oral rinse samples and 1087 saliva samples, with primer sets V3V4 (*n* = 645), V4 (*n* = 740) and V4V5 (*n* = 38) (Table S2). The RoB2 assessments were performed on these selected studies and showed the overall low risk of bias (Table S1).

### 3.2. Patient Type, 16S rRNA Amplification Primer Sets, and Study Batch Affect Overall Microbial Composition of Saliva and Oral Rinse Samples

We first evaluated the effect of primer set usage and study batch (described in the Supplementary Materials) [43,66], since specific primer regions and study batches can impact the overall microbial composition in 16S rRNA sequencing [58,68]. For saliva samples, both batch and primer effects persisted after applying MMUPHin adjustment, albeit with minor improvements (Figures S2 and S3). To minimize additional bias from primer sets [57,77,78], we analyzed each primer set separately before the subsequent analysis of saliva samples. Overall, while not completely removing batch effects, MMUPHin adjustment reduced batch effects in saliva samples (Figures S4–S9). Subsequent analyses were based on the MMUPHin adjusted datasets.

Three studies using oral rinse samples (*n* = 336) and V3V4 primers were conducted by the same research group [27,28,30]. No batch effects were observed for these V3V4 oral rinse samples, likely due to similar sample processing, collection and patient demographics (Figure S10). Thus, MMUPHin adjustment was not applied for oral rinse samples.

### 3.3. HNC Alpha Diversity Differs between Saliva and Oral Rinse

**Figure 2:**
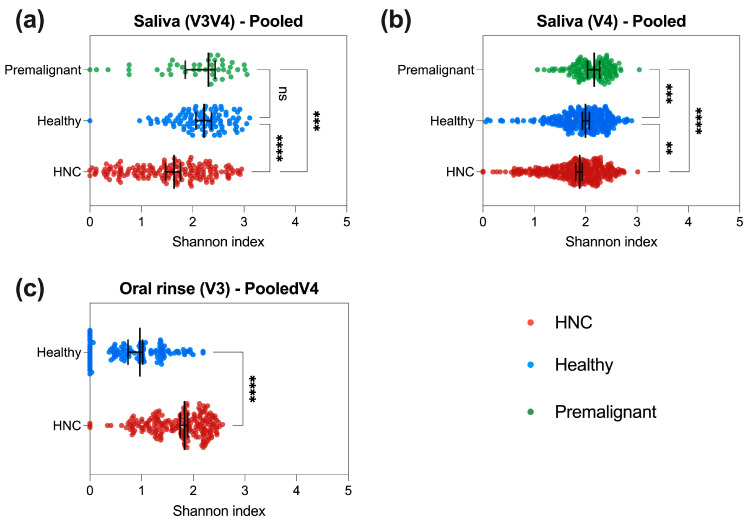
Comparison of pooled alpha diversity between HNC, premalignant and healthy patients at the genus level. Shannon diversity was measured using rarefied abundance. Shannon index for (**a**) pooled V3V4 saliva—HNC (*n* = 170), healthy (*n* = 96) and premalignant (*n* = 43); (**b**) pooled V4 saliva—HNC (*n* = 329), healthy (*n* = 282) and premalignant (*n* = 129), and (**c**) pooled V3V4 oral rinse—HNC (*n* = 206) and healthy (*n* = 130) samples. Kruskal–Wallis test with Dunn’s multiple comparison and Mann–Whitney test were performed to compare different sample groups, black line represents median. **** *p* < 0.0001, *** *p* < 0.001, ** *p* < 0.01, ns—not significant.

To determine differences in alpha diversity between patient types, Shannon index was measured for saliva and oral rinse independently (Figure 2, Figures S11 and S12). Pooled and per-batch alpha diversities were compared between patient types for each primer set (Figure 2, Figures S11 and S12). Overall, alpha diversity was different between HNC and healthy samples; however, this difference was inconsistent between saliva and oral rinse. For saliva samples, alpha diversity was higher in healthy donors (median Shannon index: V3V4 = 2.223, V4 = 1.995, V4V5 = 2.161) compared to HNC patients (median Shannon index: V3V4 = 1.640, V4 = 1.883, V4V5 = 1.914) (Figure 2a,b and Figure S11, Table S3). This finding was consistent in eight out of nine studies comparing HNC and healthy saliva samples, although some studies did not achieve statistical significance (Figure S11, Table S3). In contrast, oral rinse samples from HNC patients (median Shannon index: V3V4 = 1.832) displayed significantly higher alpha diversity than healthy donors (median Shannon index: V3V4 = 0.864) (Figure 2c, Table S4). All single studies comparing HNC and healthy oral rinse samples showed a similar trend to the pooled data (Figure S12). In this study, oral microbiome data from premalignant HNC patients were only available in saliva samples. Saliva from HNC had lower alpha diversity (median difference in Shannon index: V3V4 = −0.6688, V4 = −0.2824) compared to pre-malignant HNC saliva (Figure 2 and Figure S2), with two out of three studies displaying a same trend (Figure S11a,b). Overall, the change in alpha diversity between HNC and healthy samples varied between saliva and oral rinse, irrespective of primer set used.

### 3.4. Saliva or Oral Rinse from HNC, Premalignant and Healthy Donors Have Similar Beta Diversities at the Genus Level

**Figure 3:**
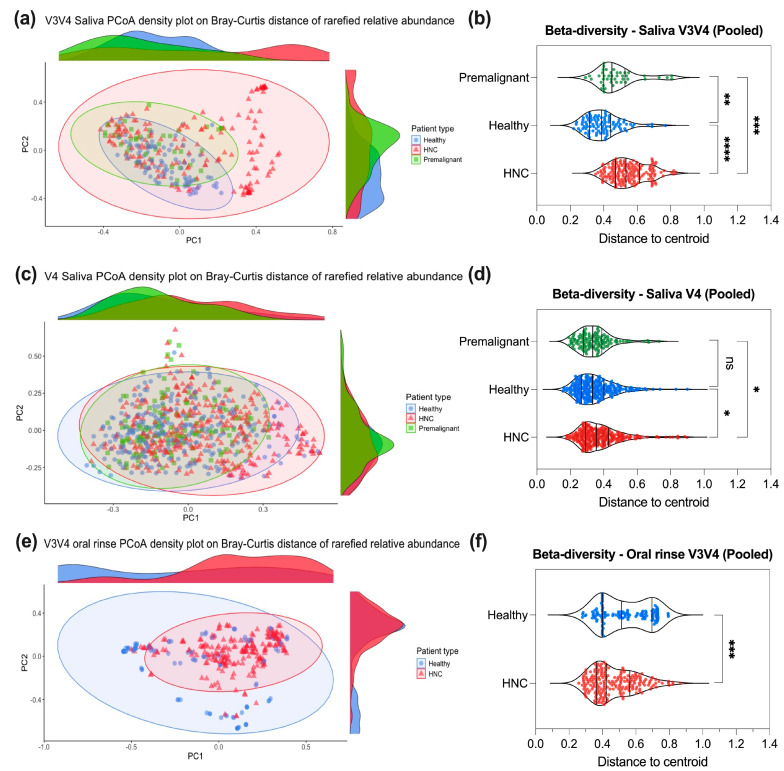
Comparison of beta diversity between HNC, premalignant and healthy patients at the genus level. Based on a conventional analysis method, raw abundance counts were rarefied and converted to relative abundance for (**a**) V3V4 saliva, (**c**) V4 saliva and (**e**) V3V4 oral rinse PCoA density plots on Bray–Curtis distance. Beta diversity for each sample was calculated as distance to centroid for each patient type for (**b**) V3V4 saliva, (**d**) V4 saliva and (**f**) V3V4 oral rinse. Kruskal–Wallis test with Dunn’s multiple comparison or Mann–Whitney test were performed to test for differences in beta diversity. **** *p* < 0.0001, *** *p* < 0.001, ** *p* < 0.01, * *p* < 0.05, ns—not significant.

To assess differences in overall microbial composition (beta diversity) between patient types for saliva or oral rinse, we used PCoA density plots of Bray–Curtis distance on rarefied relative abundance (Figure 3), PCoA plots of Euclidean distance on CLR–abundance (Figure S14), PERMANOVA tests, and differences in distance to centroid. Additionally, dispersion tests of distance to centroid for each patient type group were performed using permutest to determine if differences in dispersions exist between groups.

In saliva samples, minimal distinction between patient types was observed in the PCoA density plot based on Bray–Curtis distance with rarefied relative abundance (Figure 3a,c and Figure S13a,b). Beta diversity, measured as the distance to the group centroid, was calculated for each sample using Bray–Curtis distance. Generally, saliva samples from HNC patients (median beta diversity index: V3V4 = 0.533, V4 = 0.355, V4V5 = n.s.) showed moderately increased beta diversity index values compared to healthy (median beta diversity index: V3V4 = 0.388, V4 = 0.332, V4V5 = n.s.) and premalignant donors (median beta diversity index: V3V4 = 0.449, V4 = 0.334, V4V5 = NA) (Figure 3b,d and Figure S13b). Furthermore, a PERMANOVA test showed that the beta diversity of saliva samples was significantly different (*p* < 0.05) between HNC and healthy (PERMANOVA R^2^: V3V4 = 0.0584, V4 = 0.0175, V4V5 = 0.0779), as well as between HNC and premalignant saliva samples (PERMANOVA R^2^: V3V4 = 0.0305, V4 = 0.0238) (Table S5). However, using betadisper [74], we observed significant differences (*p* < 0.05) in dispersion between HNC and healthy, as well as between HNC and premalignant samples, indicating that differences in beta diversity were predominantly driven by differences in dispersion among groups rather than differences in their spatial medians (Table S5). Even with the compositional approach (Euclidean distance of CLR-abundance) [56], we observed similar findings whereby the beta diversity of saliva samples was largely consistent regardless of patient type (Figure S14, Table S6).

For oral rinse samples, the microbial compositions of HNC and healthy samples were largely similar (Figure 3e). A slightly greater distinction between HNC and healthy oral samples was observed in Euclidean distance PCoA density plots based on CLR-abundance (Figure S15a). Although the beta diversity of HNC oral rinse samples (median beta diversity index = 0.422) was lower than that of healthy samples (median beta diversity index = 0.515) (PERMANOVA: R^2^ = 0.0797, *p* < 0.001) (Figure 3f, Table S7), significant differences in dispersion (*p* < 0.001) were also observed between HNC and healthy samples (Table S7). Similar results were found in oral rinse samples using Euclidean distance of CLR-abundance (Figure S15, Table S8). Taken together, we found that beta diversity was largely similar across different donors, regardless of oral sampling technique used. It is likely that any differences observed were primarily driven by the variability within the patient groups (HNC, premalignant, healthy) itself.

### 3.5. Differentially Abundant Genera between HNC and Healthy Oral Samples

**Figure 4:**
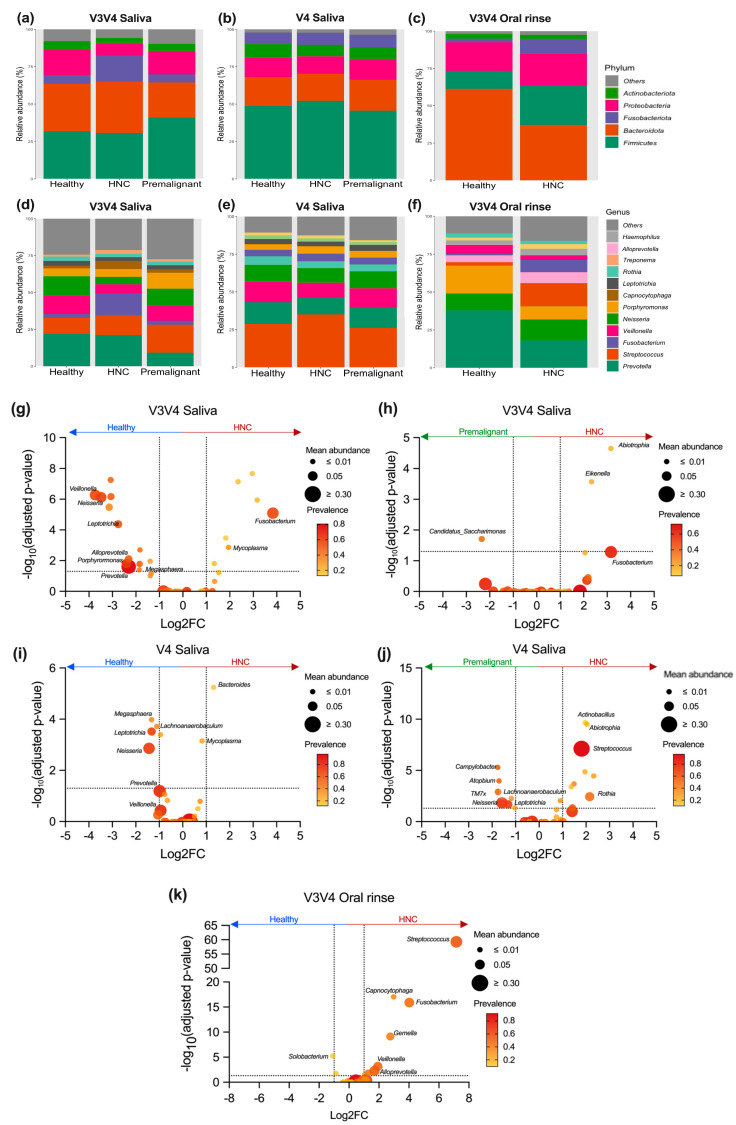
Microbial relative abundance and differential abundance analysis in saliva and oral rinse samples. Mean relative abundance of (**a**) V3V4 saliva, (**b**) V4 saliva, and (**c**) V3V4 oral rinse samples at the phylum level. Mean relative abundance of (**d**) V3V4 saliva, (**e**) V4 saliva, and (**f**) V3V4 oral rinse samples at the genus level. Differential abundance analysis (LinDA) was performed to determine differences between (**g**,**i**,**k**) HNC and healthy and (**h**,**j**) HNC and premalignant samples for (**g**,**h**) V3V4 saliva, (**i**,**j**) V4 saliva, and (**k**) V3V4 oral rinse samples. *p*-values were adjusted using the Bonferroni method.

The five most commonly detected phyla across all samples were *Firmicutes*, *Bacteroidota*, *Fusobacteriota*, *Proteobacteria*, and *Actinobacteriota* (Figure 4a–c and Figure S16a, and Table 1), consistent with a previous report [41]. However, oral rinse and saliva samples captured slightly different microbial communities. Oral rinse samples had a higher abundance of *Bacteroides* and a lower abundance of *Firmicutes* compared to saliva samples (Figure 4a–c and Figure S16a, and Table 1). At the genus level, *Streptococcus*, *Neisseria*, *Prevotella*, *Porphyromonas*, *Veillonella*, *Fusobacterium*, *Rothia* and *Treponema* were among the most prevalent across all saliva and oral rinse samples (Figure 4d–f and Figure S16b, Table 1 and Table S9a). Notable differences in relative abundance were also observed between sample type and primer sets for these genera (Figure 4d–f and Figure S16b, Table 1 and Table S9a). For instance, *Streptococcus* was detected at higher abundance using V4 or V4V5 primers than V3V4 primers in saliva samples, while the opposite trend was observed for *Prevotella*, with higher levels detected using V3V4 primers compared to V4 or V4V5 in saliva samples (Table S9a). Together, these finding suggest that the choice of primer sets and oral sampling method can significantly influence the detected microbial community, impacting the overall interpretation of the results.

We conducted differential abundance analysis using LinDA to compare differences between HNC, healthy and premalignant saliva and oral rinse samples (Figure 4g–k, Table S10) [75]. Similar to the beta diversity analysis, HNC saliva samples were more similar to healthy saliva when using V4 primers, with only seven bacterial genera showing significant differences (Figure 4i). In contrast, the V3V4 primers identified 20 bacterial genera that were significantly different between HNC and healthy saliva samples (Figure 4g). Both V3V4 and V4 primer sets consistently identified *Neisseria* (Log2FC: V3V4 = −3.48, V4 = −1.44), *Leptotrichia* (Log2FC: V3V4 = −2.76, V4 = −1.33) and *Megasphaera* (Log2FC: V3V4 = −1.86, V4 = −1.33) as enriched in healthy saliva, while *Mycoplasma* (Log2FC: V3V4 = 1.94, V4 = 0.821) was enriched in HNC saliva (Figure 4g,i, Table S10a). No significant differences were observed between HNC and healthy saliva when using V4V5 primers (Figure S16, Table S10a). Critically, the data obtained with V4V5 primers were inconsistent with those generated using V3V4 and V4 primers (Table S10a). Of note, only one study reported the use of V4V5 primers. *Fusobacterium*, a cancer-associated microbe, was enriched in HNC saliva compared to healthy saliva only when using V3V4 primers (Log2FC = 3.83, *p* < 0.05), but not when using V4 primers (Log2FC = −0.48) or V4V5 (Log2FC = −1.38). Additionally, although not statistically significant, we observed an enrichment of oral commensal bacteria (mean > 5%), such as *Prevotella* (Log2FC: V3V4 = −2.31, *p* < 0.05; V4 = −0.99, *p* > 0.05) and *Veillonella* (Log2FC: V3V4 = −3.74, *p* < 0.05; V4 = −0.95, *p* > 0.05), in HNC saliva samples when using both V3V4 and V4 primers (Table S10a). No significant differences were observed for *Streptococcus* when using either V3V4 or V4 primers (Table S10a).

Interestingly, oral rinse sampling detected fewer differentially abundant genera between HNC and healthy samples when compared to saliva sampling using the V3V4 primers. In oral rinse samples, ten bacterial genera were significantly enriched in HNC samples compared to healthy samples (Figure 4k, Table S10). Notably, *Streptococcus* (Log2FC = 7.16), *Capnocytophaga* (Log2FC = 2.97), *Fusobacterium* (Log2FC = 4.02), and *Gemella* (Log2FC = 2.75) were among the top genera elevated in HNC oral rinse (Figure 4k, Table S10b). Additionally, *Fusobacterium* and *TM7x* were the only genera showing the consistent directionality of abundance across both saliva and oral rinse samples, regardless of the primer set used (Table S10a,b). In contrast, genera such as *Veillonella*, *Alloprevotella*, and *Campylobacter* exhibited differing trends between saliva and oral rinse samples (Table S10a,b). These findings further underscore the influence of sampling technique on the results of oral microbiome studies.

Premalignant saliva samples were only available in studies that used the V3V4 and V4 primer sets, and these were compared to HNC saliva samples (Figure 4h,j, Table S10a). It is important to note that few premalignant samples datasets were available for analysis (V3V4—*n* = 43, two studies; V4—*n* = 129, one study), which limits the interpretation of the results. Nevertheless, when comparing HNC to premalignant saliva samples, *Abiotrophia* (Log2FC: V3V4 = 3.16, *p* < 0.05; V4 = 2.04, *p* < 0.05) was the only genus consistently enriched in HNC saliva (Figure 4h,j, Table S10a). Additionally, using the V4 primers, *Streptoccocus* (Log2FC = 1.81, mean abundance = 32.5%) was significantly enriched in HNC, while *Neisseria* (Log2FC = −1.58, mean abundance = 10.1%) was enriched in premalignant saliva (Figure 4h, Table S10a).

### 3.6. Classification of Oral Samples Based on sPLS-DA

Lastly, the sPLS-DA multivariate model was employed to assess whether HNC samples could be discriminated from their healthy counterparts [79]. Due to the availability of data, the only analysis utilized the V3V4 saliva, V4 saliva, and V3V4 oral rinse datasets. The area under the curve (AUC) was calculated for each donor group versus the others, serving as a metric for the model’s ability to differentiate HNC from healthy and premalignant donors. Generally, AUC values of >0.9, 0.8 to 0.9 and <0.7 represent excellent, good and poor classifier performance, respectively.

**Figure 5:**
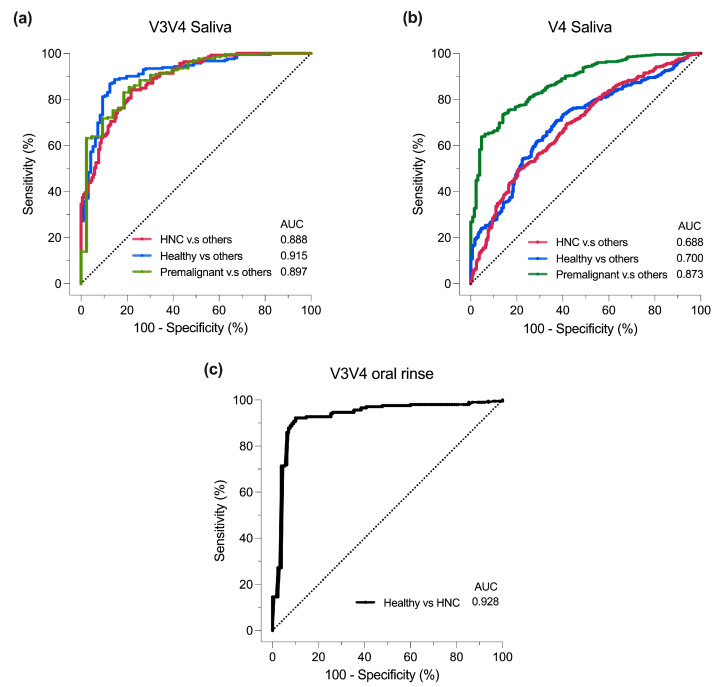
Discriminant analysis using AUROC curves based on the sPLS-DA model of the oral microbiome. AUROC curves of (**a**) V3V4 saliva, (**b**) V4 saliva and (**c**) V3V4 oral rinse. AUC was calculated for each “one vs. other” patient group and Wilcoxon test was used to test for differences between one group and others.

For saliva samples, the AUC values varied between the V3V4 and V4 primer sets. In V3V4 saliva samples, the AUC values for a donor group versus others were as follows: HNC vs. others = 0.888 (*p* < 0.05), healthy vs. others = 0.915, and premalignant vs. others = 0.897 (*p* < 0.05) (Figure 5a). In contrast, for V4 saliva samples, the AUC values were lower, as follows: HNC vs. others = 0.688 (*p* < 0.05), healthy vs. others = 0.700 (*p* < 0.05), and premalignant vs. others = 0.873 (*p* < 0.05) (Figure 5b). This suggests that when using saliva samples, the V4 primers were less effective at distinguishing HNC patients from other donor types compared to the V3V4 primers. Of note, the V3V4 saliva dataset had a smaller sample size (HNC = 170, healthy = 96, premalignant = 43) than V4 saliva samples (HNC = 329, healthy = 282, premalignant = 129) (chi-squared: *p* = 0.008), which may have resulted in the differences in AUC. Additionally, AUC may also be affected by the remaining batch effect after batch correction. For V3V4 oral rinse samples, sPLS-DA was able to discriminate HNC and healthy samples (AUC = 0.928, *p* < 0.05) (Figure 5c). Of note, all the datasets obtained for oral rinse samples originated from the same group. Overall, we found that both saliva and oral rinse sampling exhibited an ability to discriminate HNC from premalignant or healthy donors, and that this was highly dependent on the primer sets used for 16S rRNA amplification.

## 4. Discussion

There is increasing evidence that oral microbiome dysbiosis may be linked to HNC. Identifying and characterizing the key microbes that contribute to disease HNC pathobiology is an exciting avenue for the development of new diagnostics and treatment strategies. 16S rRNA microbiome sequencing has been the gold standard for microbiome analysis, and several attempts have been made to determine if this technique, when applied to oral samplings, can be developed as a non-invasive cancer diagnostic test for HNCs [8,9,10,14,16,17,24,27,28,29,30,31,32,33,34,35,42,43,44,45,46,47]. However, current studies report inconsistent findings regarding oral bacterial abundance and diversities present in HNCs in relation to healthy and premalignant donors [8,9,10,14,16,17,24,27,28,29,30,31,32,33,34,35,42,43,44,45,46,47]. In this study, we conducted a meta-analysis of 14 studies, and showed that primer set usage and oral sampling types can have a significant impact on the microbiome data, in addition to the fact that considering these key factors can lead to better discrimination between HNC, healthy and premalignant samples.

We found that alpha diversity (Shannon index) displayed a similar donor type-dependent trend across saliva samples regardless of the 16S rRNA amplification primer set used. We found that HNC patients’ saliva displayed lower richness and evenness when compared to healthy donors, corroborating previous reports [12,24,29,50,80,81,82,83,84,85]. HNC saliva displayed lower alpha diversity when compared to premalignant samples. Importantly, the overall trend of alpha diversity was consistent regardless of primer type used. Furthermore, we showed that HNC oral rinse contained greater alpha diversity than healthy oral rinse samples [28,32,47]. Unlike a previous study, which combined various oral sample types (swabs—undefined oral sites, tissues, saliva) and primer sets (V1–V3, V4, V1–V4, V3–V4, V4) in their re-analysis [55], we were able to identify statistically significant differences in alpha diversity between HNC and other donor types by separating the analysis based on sampling method [12,24,29,50,80,81,82,83,84,85]. Different sample types, even from the same patient, will display some discrepancies in microbiome detected [86]. Oral microbes are site-specialist, and bacteria species usually prefer a particular oral site and are less likely to be found at other sites [24,39,40,87]. Hence, swab-based sampling is likely to be biased due to differences in the specific sites chosen for sampling. More importantly, it is expected that intratumoural bacteria present within the tissue samples will contain a different microbiome profile [22] due to differences in environment (i.e., oxygen level) [88]. Hence, it is recommended to take caution when interpreting a combination of sample types. Moreover, a direct comparison between saliva (OMNIgene ORAL kit or fixed in Saccomanno’s fixative) and oral rinse (Scope mouthwash or nonethanol mouth wash) showed different microbial profiles from the same healthy individuals [89], supporting our study. Hence, oral sampling is critical for interpreting HNC oral microbiome data [40]**,** and it is recommended to only use one method when performing comparisons across subject groups [89].

For HNC studies [9,13,16,24,26,27,28,30,32,46,47,55,84,90], differences in beta diversity are usually measured by PERMANOVA, which holds an assumption of homogeneity in multivariate dispersion [91]. However, some of these studies did not report beta-dispersion. Our findings on beta diversity were in accordance with those from a previous meta-analysis [55], whereby scarce clustering was observed between patient types, albeit PERMANOVA analysis was statistically significant. Importantly, this study shows that the significant differences observed from PERMANOVA are primarily driven by differences in the beta dispersion of samples [91]. Based on these findings, beta diversities for saliva and oral rinse are likely similar between HNC and healthy donors, as previously reported [12,46,55].

Microbial community abundance is also affected by primer set usage and oral sampling technique [40,58]. Surprisingly, marked differences were already evident at the phylum level in V3V4 oral rinse samples. This effect was more apparent at the genus level, even for the highly abundant genera detected. Without paired saliva and oral rinse samples from the same patient to benchmark, one cannot exclude interpatient or study differences causing this phenomenon. At the genus level, we observed similarities to previous reports where *Neisseria* [9,15,81,83,92], *Leptotrichia* [81,83], and *Megasphaera* [26] were elevated in healthy saliva, while *Mycoplasma* [17] was elevated in HNC saliva. We also found contradictory results on the abundances for *Neisseria* [17,24,29,50] and *Leptotrichia* [17,24]. Importantly, we believe that our findings are more robust, because of our significantly larger sample sizes and the consistency of our results from both V3V4 and V4 primer set data. Although limited to small sample sizes in premalignant saliva samples, we also identified consistent elevation in *Abiotrophia* in HNC saliva when compared to premalignant saliva samples.

In contrast to previous studies [26,81,83], our meta-analysis results suggest that *Streptococcus* abundance is similar between HNC and healthy saliva samples. *Streptococcus* is enriched in HNC tumor tissues, with species-specific effects such as tumor growth promotion (*S. mutans*) and CD8+ T cell activation (*S. anginosus*, *S. mitis*, *S. salivarius*) [43]. Additionally, *Neisseria* species *N. flavescens* and *N. sicca* can induce HNC necroptosis and pyroptosis in vitro and in vivo, respectively [93,94]. However, *N. sicca* can also suppress tumor inflammation by downregulating NF-kB and IL-6 [93]. This highlights the complexity of the oral microbiome, the limitations of genera-level identification, and the need for higher taxonomical resolution for subsequent studies on the oral microbiome’s impact on HNC disease progression [72,95].

For V3V4 oral rinse samples, there were a total of 10 genera significantly elevated in HNC patients. When compared to V3V4 saliva samples, *Fusobacterium* and *TM7x* were the only genera that was consistent across both sampling techniques, while contrary to this result, *Veillonella*, *Alloprevotella*, and *Campylobacter* were lower in HNC saliva. The remaining five bacterial genera identified in oral rinse samples were not significantly different across donors in V3V4 saliva. Taken together with our alpha diversity findings, we reveal that sampling technique is critical for oral microbiome analysis. The interpretation of the results of oral microbiome studies, including systematic reviews and meta-analyses that combine different sampling techniques, should be viewed with this limitation in mind [38,53,55].

Finally, we used the sPLS-DA model [76] to assess 16S rRNA sequencing oral microbiome profiling’s ability to classify different HNC disease states. We found varying discriminating potential between V3V4 and V4 saliva datasets, with the V3V4 saliva dataset being most similar to previous studies, with AUCs within similar ranges in saliva (AUC = 0.63–0.95) and oral rinse (AUC = 0.62–0.98) samples [12,13,20,21,27,30,32,33,34,48,49,50]. Additionally, since all the oral rinse data originate from the same research group, more studies are required to determine if there are geographical differences that are currently not captured by this dataset.

This comprehensive study on saliva and oral rinse samples in HNC highlights the use of short-read 16S rRNA sequencing microbiome analysis as a potential clinical diagnostic. It is crucial to recognize the inherent limitations of this study. While MMUPHin batch correction was employed to alleviate batch effects, it is insufficient to eradicate batch effects completely. Most batch correction tools are designed to mitigate or alleviate technical variations rather than being modeled particularly on specific sources (i.e., differences in extraction protocols, primers, amplicon regions amplified) [57,68,78]. Moreover, batch effects can give rise to misleading interpretations in machine learning models [96]. The interpretability of machine learning (ML) models, which function as a “black box”, remains a key limitation due to the lack of a clear biological understanding of features [96]. Thus, incorporating prior knowledge on features into current models may address these issues [96]. Furthermore, we cannot rule out the idea that batch effects may reflect geographical differences, which include lifestyle factors such as diet, which is known to affect oral microbial diversity [41]. Additionally, this study may be improved by increasing the sample size. This may be achieved by including all publicly available oral samples such as swabs and excluding saliva or oral rinse samples, so as to provide a larger cohort and range of sample types. However, limited available metadata in the excluded studies, such as those regarding the specific site of swab taken and treatment (therapeutic and antibiotic usage), will skew overall interpretations if included. In addition, we do not have full clinical metadata, such as TNM staging, which can provide better insights into whether certain microbiome profiles may have discriminatory and prognostic potential [32]. For instance, *Fusobacterium periodonticium*, *Parvimonas micra*, *Streptococcus constellatus*, and *Filifactor alocis* showed good discriminatory potential (AUC > 0.8) for stage 4 oral squamous cell carcinoma [32].

Conventionally, bacterial 16S rRNA sequencing is performed by amplifying a particular variable region of the 16S rRNA gene (V3–V4, V4, V4–V5, V6–V8 regions), using short-read sequencing technologies [58]. As shown in this study, differences in 16S rRNA region and primer amplification bias can skew the overall microbial community leading to misinterpretations of the underlying biology [58]. Moreover, short-read 16S rRNA sequencing is limited to genus-level resolution due to the 16S rRNA gene coverage [43,72,95]. With advancements in long-read sequencing technologies, one option is to sequence the whole 16S rRNA gene, which can provide species-level taxonomical coverage while minimizing primer bias if optimized and benchmarked well [72,95]. Alternatively, metagenomics, a more costly approach, can be used to avoid amplification and primer biasness, while providing microbial genome-level resolution if sequenced with ample depth [43,72,95]. Currently, HNC is commonly diagnosed after the tumor has significantly progressed, necessitating the need for cost-effective, accurate, and rapid diagnostic tools for HNCs [97]. Virome’s CancerDetect^®^ Oral & Throat kit (Virome Life Sciences, NY, USA) is the only commercial non FDA-approved saliva-based oral and oropharyngeal cancer detection kit available, and is based on profiling the microbial transcriptome [97]. We propose that our study will aid in the development of microbial genomic-based tools for HNC diagnostics.

## 5. Conclusions

In conclusion, this study highlights the current state of using oral microbial genomics as a diagnostic tool for HNCs. Oral sampling (saliva and oral rinse) and primer set usage in short-read 16S rRNA sequencing are key determinants in the data generated, and are the likely cause of discrepancies observed in the field. Critically, the core microbiome and overall microbial composition of saliva and oral rinse samples were largely similar. As a diagnostic tool, using short-read 16S rRNA sequencing alone would be inadequate, justifying the adoption of cutting-edge microbial screening methods such as long-read 16S rRNA sequencing or metagenomics.

## Figures and Tables

**Table 1:** Mean relative abundance of core phylum and genera across saliva and oral rinse samples.

Phylum	Saliva Relative Abundance (Mean ± SD)	Oral Rinse Relative Abundance (Mean ± SD)
*Firmicutes*	V3V4 = 32.4 ± 23.4% V4 = 49.7 ± 19.0% V4V5 = 43.2 ± 14.5%	V3V4 = 20.9 ± 20.4%
*Bacteroidota*	V3V4 = 32.2 ± 27.2% V4 = 19.1 ± 14.3% V4V5 = 33.0 ± 14.8%	V3V4 = 46.5 ± 29.5%
*Fusobacteriota*	V3V4 = 12.2 ± 21.0% V4 = 8.00 ± 9.01% V4V5 = 2.93 ± 2.82%	V3V4 = 7.06 ± 11.3%
*Proteobacteria*	V3V4 = 12.0 ± 16.0% V4 = 12.4 ± 11.5% V4V5 = 16.2 ± 13.8%	V3V4 = 20.3 ± 24.2%
*Actinobacteriota*	V3V4 = 4.15 ± 7.78% V4 = 8.32 ± 7.64% V4V5 = 3.29 ± 3.46%	V3V4 = 2.90 ± 5.79%
**Genera**	**Saliva** **Relative Abundance (Mean ± SD)**	**Oral Rinse** **Relative Abundance (Mean ± SD)**
*Streptococcus*	V3V4 = 13.4 ± 17.4% V4 = 31.1 ± 18.1% V4V5 = 29.3 ± 16.2%	V3V4 = 10.3 ± 15.4%
*Neisseria*	V3V4 = 8.38 ± 13.8% V4 = 10.5 ± 10.7% V4V5 = 9.17 ± 7.65%	V3V4 = 12.7 ± 19.4%
*Prevotella*	V3V4 = 19.6 ± 21.9% V4 = 13.0 ± 12.5% V4V5 = 13.1 ± 9.35%	V3V4 = 25.8 ± 28.1%
*Porphyromonas*	V3V4 = 6.05 ± 13.4% V4 = 4.40 ± 7.04% V4V5 = 12.9 ± 9.89%	V3V4 = 12.5 ± 21.6%
*Veillonella*	V3V4 = 8.78 ± 10.8% V4 = 11.6 ± 11.5% V4V5 = 4.84 ± 4.93%	V3V4 = 4.1 ± 9.2%

## Data Availability

No new sequencing data were generated, and the BioProject of all sequencing data can be found in Supplementary Document.

## Supplementary Materials

The following supporting information can be downloaded at: https://www.mdpi.com/article/10.3390/pathogens13100826/s1, Figure S1: Workflow of study; Figure S2: PCoA density plot on Euclidean distance of CLR-abundance for all saliva; Figure S3: PCoA density plot on Bray–Curtis distance of rarefied relative abundance for all saliva; Figure S4: V3V4 saliva samples for unadjusted and after MMUPHin adjustment based on CLR-abundance; Figure S5: V3V4 saliva samples unadjusted and after MMUPHin adjustment based on rarefied relative abundance; Figure S6: V4 saliva samples unadjusted and after MMUPHin adjustment based on CLR-abundance; Figure S7: V4 saliva samples unadjusted and after MMUPHin adjustment based on rarefied relative abundance; Figure S8: V4V5 saliva samples unadjusted and after MMUPHin adjustment based on CLR-abundance; Figure S9: V4V5 saliva samples unadjusted and after MMUPHin adjustment based on rarefied relative abundance; Figure S10: PCoA density plot for V3V4 unadjusted oral rinse samples; Figure S11: Comparison of alpha diversity per study for HNC, premalignant and healthy saliva samples; Figure S12: Comparison of alpha diversity per study for HNC, premalignant and healthy oral rinse samples; Figure S13: Comparison of V4V5 saliva beta diversity between HNC, premalignant and healthy patients at the genus level; Figure S14: Comparison of saliva beta diversity between HNC, premalignant and healthy patients at the genus level based on CLR abundance; Figure S15: Comparison of oral rinse beta diversity between HNC and healthy patients at the genus level based on CLR abundance; Figure S16: Microbial relative abundance and differential abundance analysis for V4V5 saliva samples; Table S1: Risk of bias and search terms; Table S2: Samples information and study batch post filtering and processing; Table S3: Alpha diversity analysis statistics for saliva samples; Table S4: Alpha diversity analysis statistics for oral rinse samples; Table S5: Beta diversity analysis for saliva based on rarefied relative abundance; Table S6: Beta diversity analysis for saliva based on CLR abundance; Table S7: Beta diversity analysis for oral rinse based on rarefied relative abundance; Table S8: Beta diversity analysis for oral rinse based on CLR abundance; Table S9: Relative abundance for saliva and oral rinse; Table S10: Differential abundance analysis for saliva and oral rinse samples. File S1 contains PRISM 2020 checklist [98]. File S2 describes batch adjustment and evaluation (Figures S2–S10) [46,57,66,68,77,78].
