# Supplementary material for: Is Short-Read 16S rRNA Sequencing of Oral Microbiome Sampling a Suitable Diagnostic Tool for Head and Neck Cancer?
Source: Pathogens. 2024 Sep 24;13(10):826. doi: 10.3390/pathogens13100826 (PMC11510575; doi:10.3390/pathogens13100826)
Supplement: Supplementary file 1 [file pathogens-13-00826-s001.zip › File S2.pdf]

Appendix B

1. Batch-correction for saliva samples

Batch effect was primarily assessed using principal coordinate analysis (PCoA) density plot, and PERMANOVA on two methods of overall microbial composition (beta-diversity): 1) CLR-normalized abundance (Euclidean distance), 2) Rarefied relative abundance (Bray-Curtis distance) [1]. Additional assessments were also performed using heatmap clustering analysis, alignment score, partial redundancy analysis (pRDA), and R^2 assessment, as described previously [2, 3]. Alignment score is used to evaluate if samples from different study batches were better mixed after batch correction [2, 3]. The score ranges from 0 to 1, with scores closer to 1 being good mixing and 0 being poor mixing. Partial redundancy analysis (pRDA) was performed to calculate the proportion of variance explained by patient type, batch effects and their intersection. For an unbalanced batch X patient type design, intersection between batch and patient type is expected. For a balanced batch X patient type design, intersection between batch and patient type is expected to be null. Lastly, the variance explained of batch and patient type for each microbial variable were measured using R^2 values were estimated by one-way ANOVA with either batch or patient type effect as covariate.

1A) All saliva samples (Figure S2 – S3)

First, we analysed all saliva samples obtained from different primer (V3V4, V4, V4V5) and study batch (BioProject) together, to assess if these factors would affect overall microbial compositions before and after MMUPHin adjustment (Figure S2). Overall, beta-diversity PCoA plot of all saliva samples based on Euclidean distance of CLR-abundance or Bray-Curtis distance of rarefied relative abundance, showed that saliva samples clusters similarly before and after MMUPHin adjustment, regardless of patient types (Figure S2c, S2d). Primer effects were marginally alleviated, with substantial differences in clustering between primers within each patient type (Figure S2c, S2d, S3c, S3d). Using PERMANOVA, significant batch and primer effects were observed ($p < 0.001$) for Euclidean distance of CLR-abundance ($R^2_{(Study\ batch)} = 0.19216$, $R^2_{(Primer)} = 0.03368$, $R^2_{(Patient\ type)} = 0.00728$) and Bray-Curtis distance of rarefied relative abundance ($R^2_{(Study\ batch)} = 0.23950$, $R^2_{(Primer)} = 0.06834$, $R^2_{(Patient\ type)} = 0.00665$). After applying MMUPHin, we observed an improvement in batch effect and primer effects were observed for Euclidean distance of CLR-abundance ($R^2_{(Study\ batch)} = 0.17450$, $R^2_{(Primer)} = 0.02347$, $R^2_{(Patient\ type)} = 0.00732$) and Bray-Curtis distance of rarefied relative abundance ($R^2_{(Study\ batch)} = 0.15875$, $R^2_{(Primer)} = 0.02745$, $R^2_{(Patient\ type)} = 0.00698$), albeit studies still display significant differences ($p < 0.001$) based on study batches. Targeting short amplicons of 16S rRNA using different primers is known to result in differences in coverage and influences the microbial composition [4]. Most batch-correction tools are designed to mitigate or alleviate technical variations rather than being modelled particularly to specific sources (i.e. differences in extraction protocols, primers, amplicon regions amplified) [1, 5, 6]. To minimize potential biasness introduced by primer sets, we opt to analyse each primer sets separately for subsequent analysis [4-6]. For each primer set (for studies with more than one study batch), MMUPHin was performed to correct for batch effects, and additional assessments were performed to compare before and after MMUPHin adjustment [2, 3].

1B) V3V4 saliva (Figure S4 – S5)

There were 7 studies using V3V4 for saliva samples. Using PERMANOVA, significant batch effects were observed ($p < 0.001$) for Euclidean distance of CLR-abundance ($R^2 = 0.230$) and Bray-Curtis distance of rarefied relative abundance ($R^2_{(Study\ batch)} = 0.301$). After applying

MMUPHin, we observed an improvement in batch effect for both Euclidean distance of CLR-abundance ($R^2_{(Study\ batch)}$ = 0.211) and Bray-Curtis distance of rarefied relative abundance ($R^2_{(Study\ batch)}$ = 0.204), albeit studies still display significant differences ($p < 0.001$) based on study batches.

Similarly, additional assessment also showed slight improvements in batch effects after MMUPHin adjustment. Overall sum of R^2 of all microbial variables (Figure S4e and S5e) and R^2 of each microbial variable (Figure S4f and S5f) estimated by one-way ANOVA with study batch as covariate, improved after MMUPHin correction, for both CLR-abundance (*Overall sum of R^2*: Unadjusted = 18.859, MMUPHin = 17.897, *Mean R^2 of each microbial variable*: Unadjusted = 0.222, MMUPHin = 0.211) and rarefied relative abundance (*Overall sum of R^2:* Unadjusted = 7.053, MMUPHin = 5.360, *Mean R^2 of each microbial variable*: Unadjusted = 0.081, MMUPHin = 0.059). Correspondingly, pRDA assessment (Figure S4h and S5h) also showed similar improvement after MMUPHin correction for batch variance for both CLR-abundance (Unadjusted = 0.162, MMUPHin = 0.147) and rarefied relative abundance (Unadjusted = 0.054, MMUPHin = 0.031). Lastly, alignment score suggest that samples were more well-mixed after MMUPHin adjustment for both CLR-abundance (Unadjusted = 0.435, MMUPHin = 0.461) and rarefied relative abundance (Unadjusted = 0.526, MMUPHin = 0.635) (Figure S4g and S5g).

1C) V4 Saliva (Figure S6 – S7)

There were 5 studies using V4 primer set for saliva samples. Using PERMANOVA, significant batch effects were observed ($p < 0.001$) for Euclidean distance of CLR-abundance ($R^2 = 0.170$) and Bray-Curtis distance of rarefied relative abundance ($R^2 = 0.211$). After applying MMUPHin, we observed an improvement in batch effect for both Euclidean distance of CLR-abundance ($R^2 = 0.147$) and Bray-Curtis distance of rarefied relative abundance ($R^2 = 0.108$), albeit studies still display significant differences ($p < 0.001$) based on study batches. Similarly, additional assessment also showed slight improvements in batch effects after MMUPHin adjustment.

Similarly, additional assessment also showed slight improvements in batch effects after MMUPHin adjustment. Overall sum of R^2 of all microbial variables (Figure S6e and S7e) and R^2 of each microbial variable (Figure S6f and S7f) estimated by one-way ANOVA with study batch as covariate, improved after MMUPHin correction, for both CLR-abundance (*Overall sum of R^2*: Unadjusted = 14.470, MMUPHin = 13.523, *Mean R^2 of each microbial variable*: Unadjusted = 0.168, MMUPHin = 0.157) and rarefied relative abundance (*Overall sum of R^2:* Unadjusted = 4.098, MMUPHin = 2.887, *Mean R^2 of each microbial variable*: Unadjusted = 0.0482, MMUPHin = 0.0340). Correspondingly, pRDA assessment (Figure S4h and S5h) also showed similar improvement after MMUPHin correction for batch variance for both CLR-abundance (Unadjusted = 0.141, MMUPHin = 0.133) and rarefied relative abundance (Unadjusted = 0.040, MMUPHin = 0.026). Lastly, alignment score suggest that samples were more well-mixed after MMUPHin adjustment for both CLR-abundance (Unadjusted = 0.308, MMUPHin = 0.339) and rarefied relative abundance (Unadjusted = 0.406, MMUPHin = 0.522) (Figure S6g and S7g).

1D) V4V5 Saliva (Figure S8 – S9)

There were only 2 studies (PRJNA412445 and PRJNA722880) using V4V5 primer set for saliva samples. V4V5 primer set consist of smaller sample size (n = 55) and study batch

PRJNA412445 only contained HNC saliva samples. Nevertheless, we applied MMUPHin adjustment and also assessed for batch effects (Figure S8 – S9). Using PERMANOVA, significant batch effects were observed ($p < 0.001$) for Euclidean distance of CLR-abundance ($R^2 = 0.270$) and Bray-Curtis distance of rarefied relative abundance ($R^2 = 0.368$). After applying MMUPHin, we observed an improvement in batch effect for Euclidean distance of CLR-abundance ($R^2 = 0.252$) and Bray-Curtis distance of rarefied relative abundance ($R^2 = 0.221$), albeit studies still display significant differences ($p < 0.001$) based on study batches. These batch differences in HNC V4V5 saliva samples can also be observed in the PCoA plots (Figure S8 and S9).

Additional assessments were also performed to evaluate batch adjustment. Overall sum of R^2 of all microbial variables (Figure S8e and S9e) and R^2 of each microbial variable (Figure S8f and S9f) estimated by one-way ANOVA with study batch as covariate, slightly improved after MMUPHin correction, for both CLR-abundance (*Overall sum of* R^2: Unadjusted = 17.831, MMUPHin = 16.837, *Mean* R^2 *of each microbial variable*: Unadjusted = 0.251, MMUPHin = 0.237) and rarefied relative abundance (*Overall sum of* R^2*:* Unadjusted = 5.207, MMUPHin = 4.200, *Mean* R^2 *of each microbial variable*: Unadjusted = 0.0777, MMUPHin = 0.0627). Correspondingly, pRDA assessment also showed similar improvement after MMUPHin correction for batch variance (Unadjusted = 0.126, MMUPHin = 0.121). However, there were null treatment variance observed in pRDA assessment. Lastly, alignment score suggest that samples were only slightly mixed for rarefied relative abundance (Unadjusted = 0.120, MMUPHin = 0.163), with no changes found for CLR-abundance (Figure S8g and S9g). Since V4V5 primer sets only contained two studies, with one study only containing HNC saliva samples, these studies were not included in main manuscript due to lack of samples. Furthermore, based on our assessment, batch effects were prominent even after MMUPHin adjustment, whereby study batch PRJNA412445 HNC saliva sample clustered away from PRJNA722880 HNC saliva samples. Hence, further analysis was only performed on PRJNA722880 in for V4V5 saliva primer set.

2. Batch correction for oral rinse samples (Figure S10)

Batch correction and assessments were performed similarly as mentioned. For oral rinse samples, there were a total of three studies (V3V4 primers: n = 336 (3 studies)). Of note, the remaining three studies using V3V4 oral rinse samples were from the same laboratory group. Using PERMANOVA, we found no significant differences ($p > 0.05$) in study batch effects. Similarly, no significant clustering based on batch was observe on PCoA density plots (Figure S10). Since these samples were from the same study laboratory, it is likely that sample processing, collections, and patient demographics were similar for these samples, resulting in no significant differences in in batch effects. Therefore, we used the uncorrected datasets for oral rinse V3V4 oral rinse samples.

References

1. Ma S, Shungin D, Mallick H *et al*. Population structure discovery in meta-analyzed microbial communities and inflammatory bowel disease using MMUPHin. *Genome Biology* 23(1), 208 (2022).
2. Yeo K, Li R, Wu F *et al*. Identification of consensus head and neck cancer-associated microbiota signatures: a systematic review and meta-analysis of 16S rRNA and The Cancer Microbiome Atlas datasets. *J Med Microbiol* 73(2), (2024).
3. Wang Y, Ka LC. PLSDA-batch: a multivariate framework to correct for batch effects in microbiome data. *Brief Bioinform* 24(2), (2023).
4. Abellan-Schneyder I, Matchado MS, Reitmeier S *et al*. Primer, Pipelines, Parameters: Issues in 16S rRNA Gene Sequencing. *mSphere* 6(1), (2021).
5. Gibbons SM, Duvallet C, Alm EJ. Correcting for batch effects in case-control microbiome studies. *PLoS Comput. Biol.* 14(4), e1006102 (2018).
6. Nearing JT, Comeau AM, Langille MGI. Identifying biases and their potential solutions in human microbiome studies. *Microbiome* 9(1), 113 (2021).
