# Supplementary material for: Is Short-Read 16S rRNA Sequencing of Oral Microbiome Sampling a Suitable Diagnostic Tool for Head and Neck Cancer?
Source: Pathogens. 2024 Sep 24;13(10):826. doi: 10.3390/pathogens13100826 (PMC11510575; doi:10.3390/pathogens13100826)
Supplement: Supplementary file 1 [file pathogens-13-00826-s001.zip › Supplementary Materials.pdf]

Supplementary Figures

Figure S1. Study analysis workflow.

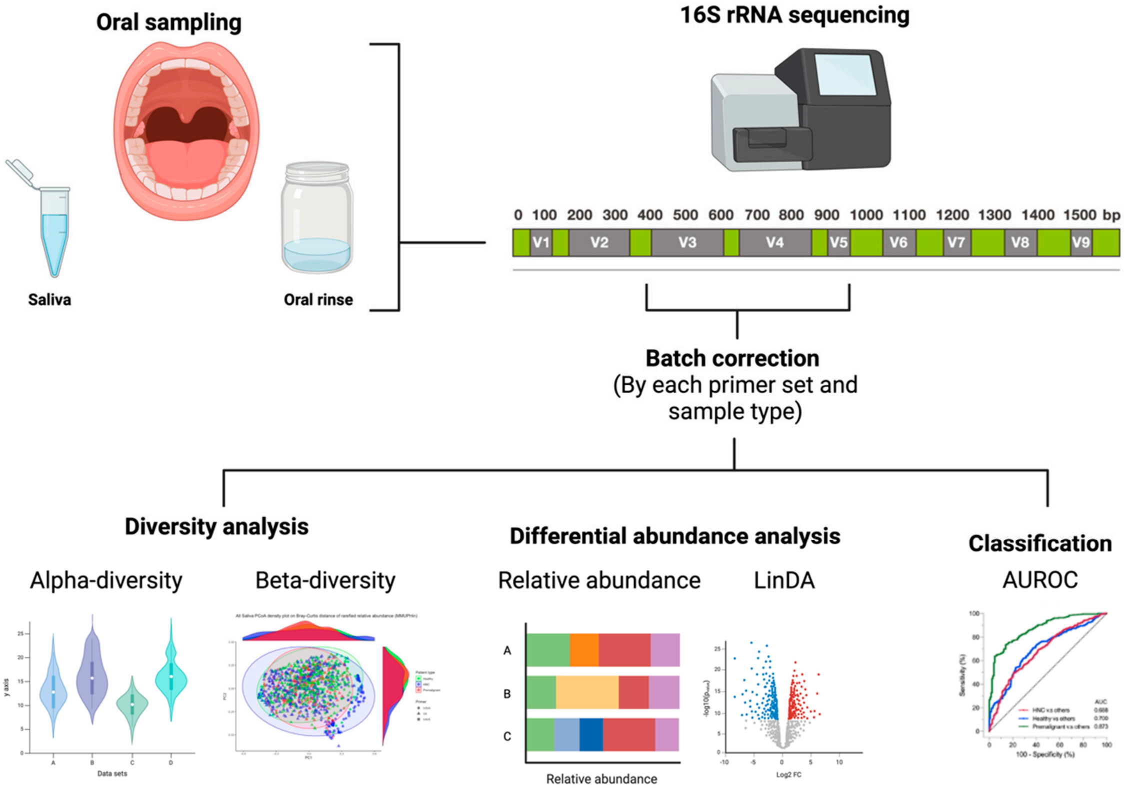

Figure S1: Workflow of study. Saliva and oral rinse 16S rRNA data were downloaded and batch correction was applied as per primer set and sample type. Diversity analysis, differential abundance analysis and classification analysis were applied on batch corrected datasets

(a)

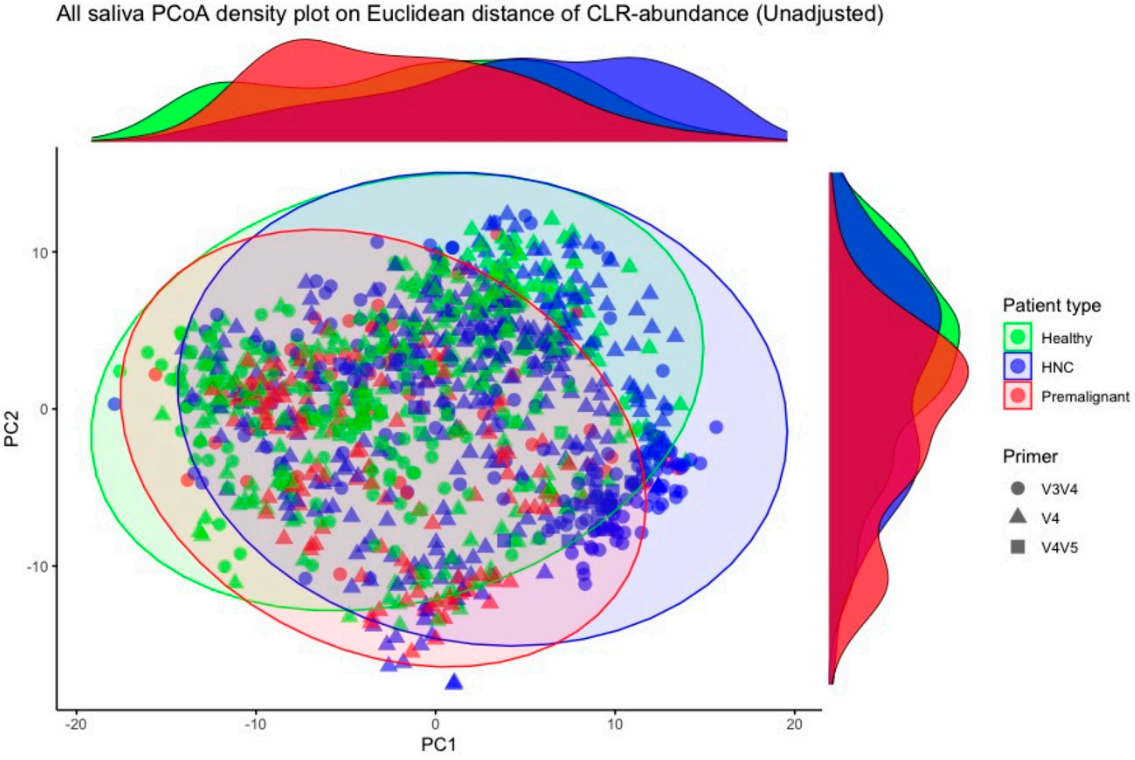
All saliva PCoA density plot on Euclidean distance of CLR-abundance (Unadjusted)
PC2
PC1
10
0
-10
-20
-10
0
10
20
Patient type
Healthy
HNC
Premalignant
Primer
V3V4
V4
V4V5

(b)

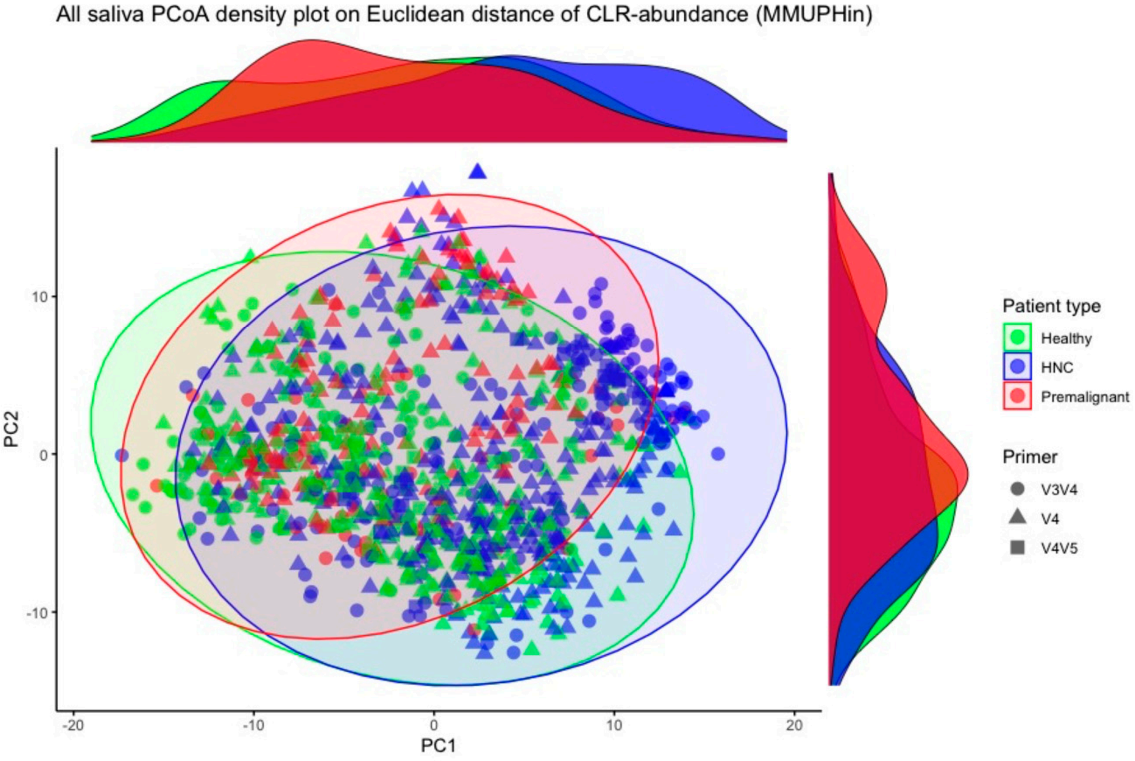
All saliva PCoA density plot on Euclidean distance of CLR-abundance (MMUPHin)
PC2
PC1
10
0
-10
-20
-10
0
10
20
Patient type
Healthy
HNC
Premalignant
Primer
V3V4
V4
V4V5

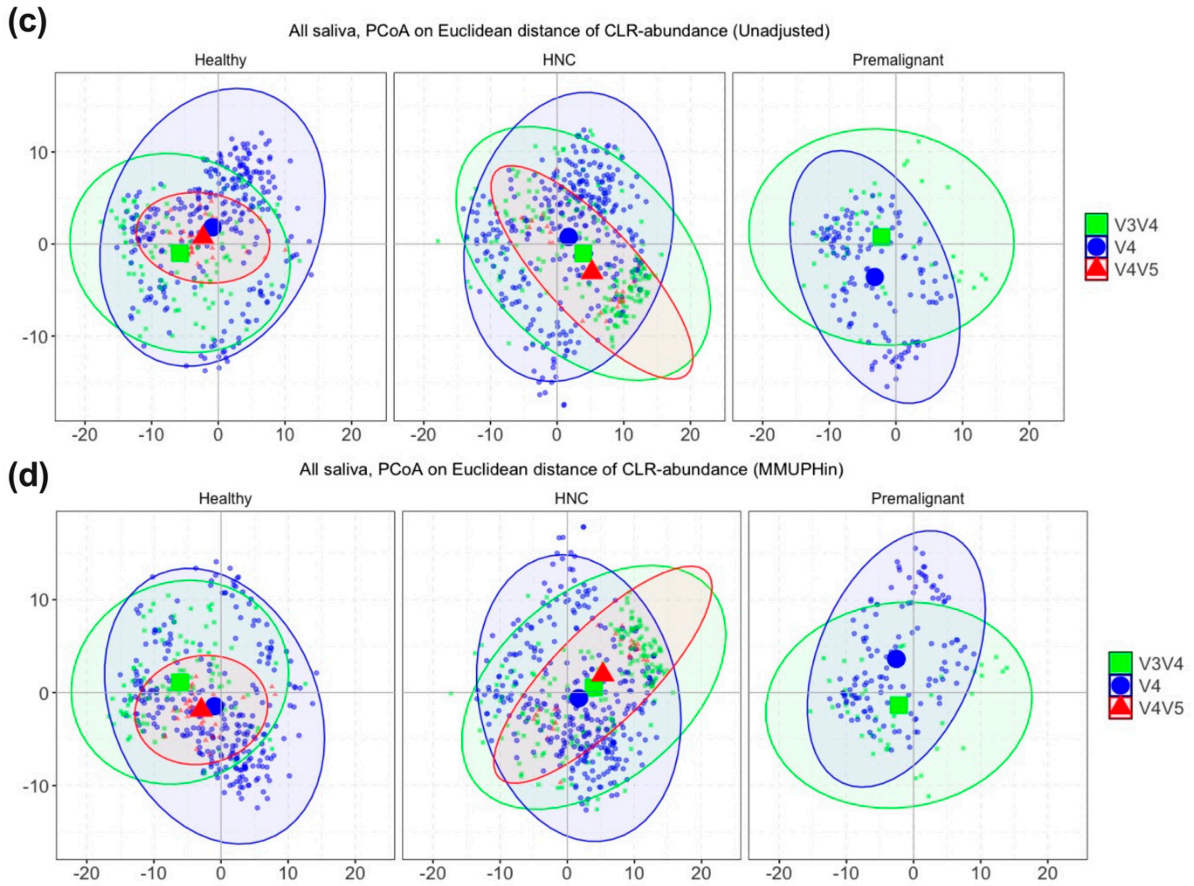

Figure S2: PCoA density plot on Euclidean distance of CLR-abundance for all saliva (HNC = 508, healthy = 407, premalignant = 172). Based on CoDA method, raw abundance counts were converted to CLR-abundance (offset = 0.5), and CLR-abundance were used for each plot. (a) Unadjusted and (b) MMUPHin-adjusted PCoA density plots facet by patient type. (c) Unadjusted and (d) MMUPHin-adjusted PCoA density plot for Healthy, HNC, and Premalignant saliva samples facet by primers.

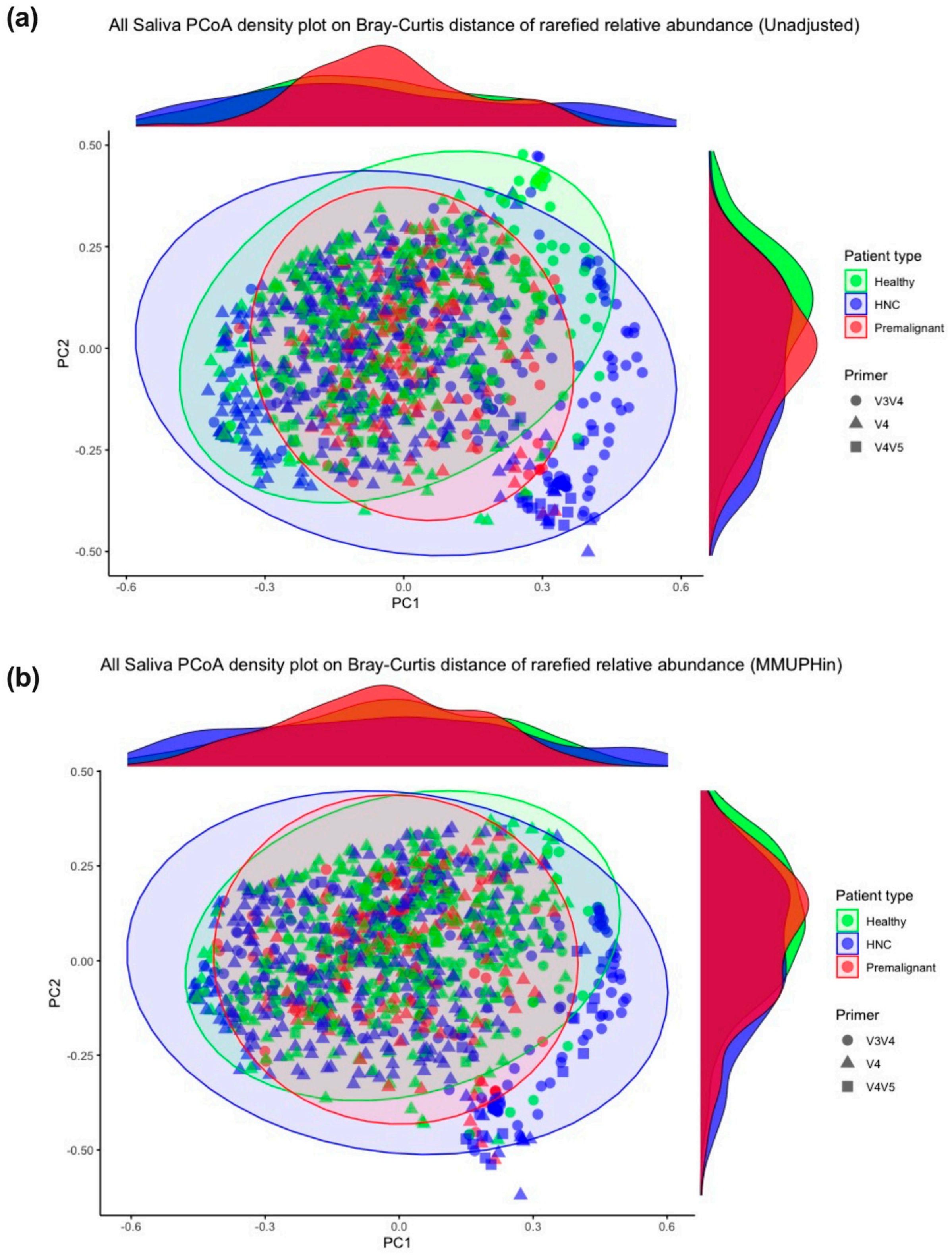
(a)
All Saliva PCoA density plot on Bray-Curtis distance of rarefied relative abundance (Unadjusted)
PC2
PC1
0.50
0.25
0.00
-0.25
-0.50
-0.6
-0.3
0.0
0.3
0.6
Patient type
Healthy
HNC
Premalignant
Primer
V3V4
V4
V4V5
(b)
All Saliva PCoA density plot on Bray-Curtis distance of rarefied relative abundance (MMUPHin)
PC2
PC1
0.50
0.25
0.00
-0.25
-0.50
-0.6
-0.3
0.0
0.3
0.6
Patient type
Healthy
HNC
Premalignant
Primer
V3V4
V4
V4V5

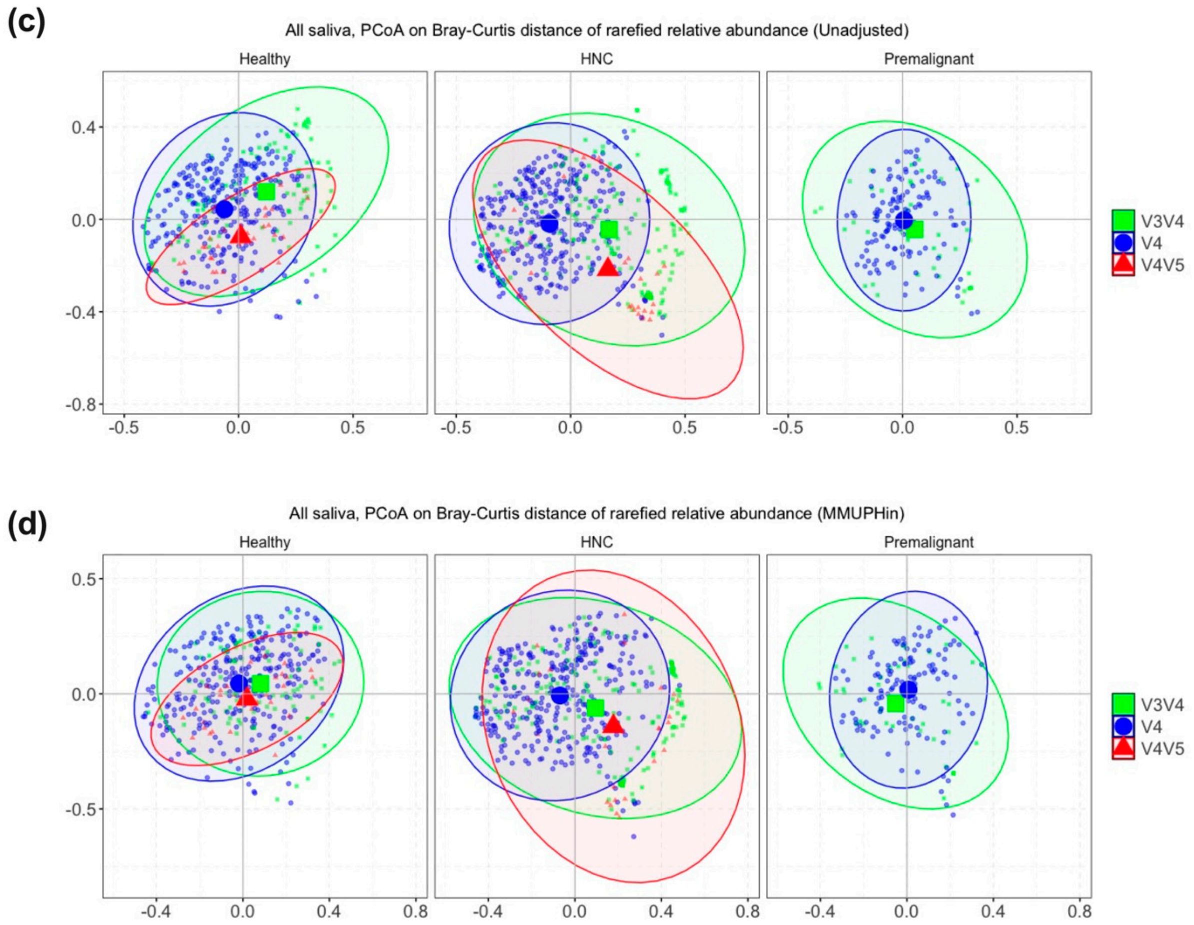

Figure S3: PCoA density plot on Bray-Curtis distance of rarefied relative abundance for all saliva (HNC = 508, healthy = 407, premalignant = 172). Based on conventional analysis method, raw abundance counts were rarefied and converted to relative abundance for each plot. (a) Unadjusted and (b) MMUPHin-adjusted PCoA density plots facet by patient type. (c) Unadjusted and (d) MMUPHin-adjusted PCoA density plot for healthy, HNC, and premalignant saliva samples facet by primers.

(a)

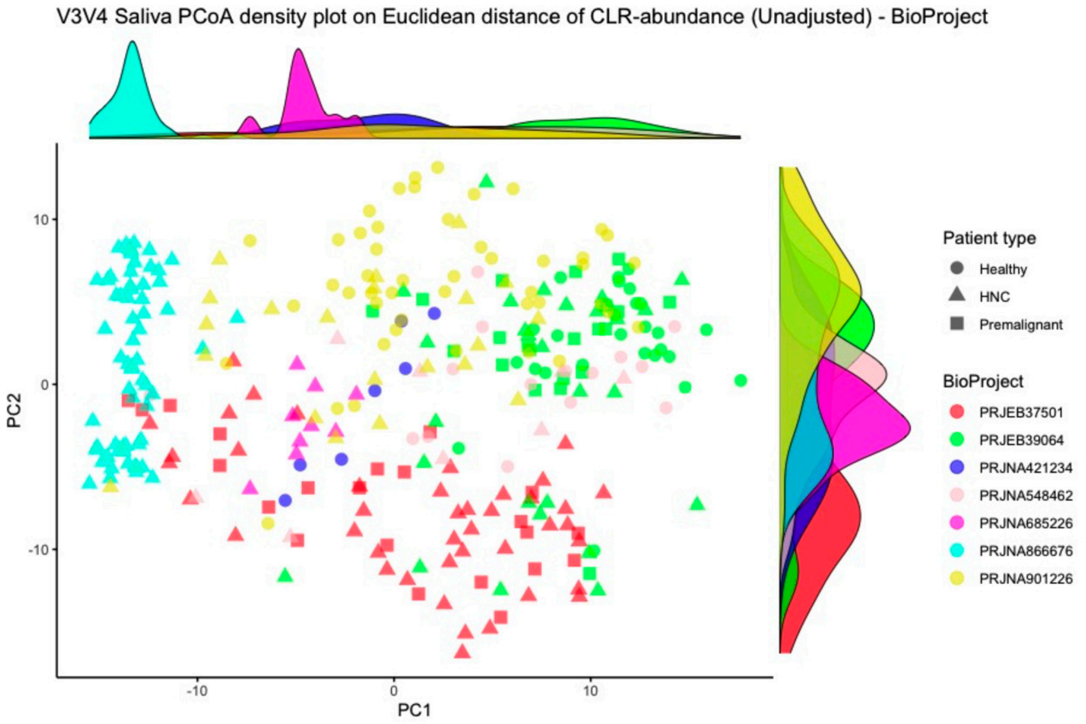

V3V4 Saliva PCoA density plot on Euclidean distance of CLR-abundance (Unadjusted) - BioProject
PC2
PC1
10
0
-10
-10
0
10
Patient type
Healthy
HNC
Premalignant
BioProject
PRJEB37501
PRJEB39064
PRJNA421234
PRJNA548462
PRJNA685226
PRJNA866676
PRJNA901226

(b)

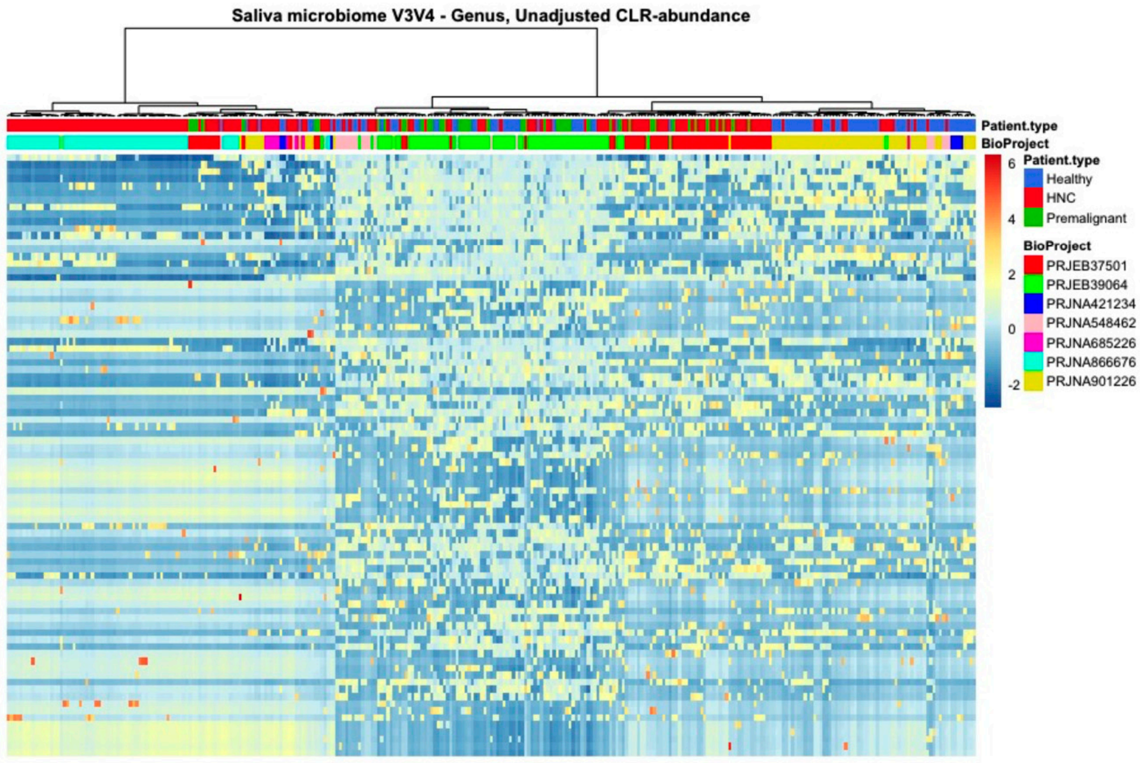

Saliva microbiome V3V4 - Genus, Unadjusted CLR-abundance
Patient.type
BioProject
6
4
2
0
-2
Patient.type
Healthy
HNC
Premalignant
BioProject
PRJEB37501
PRJEB39064
PRJNA421234
PRJNA548462
PRJNA685226
PRJNA866676
PRJNA901226

(c)

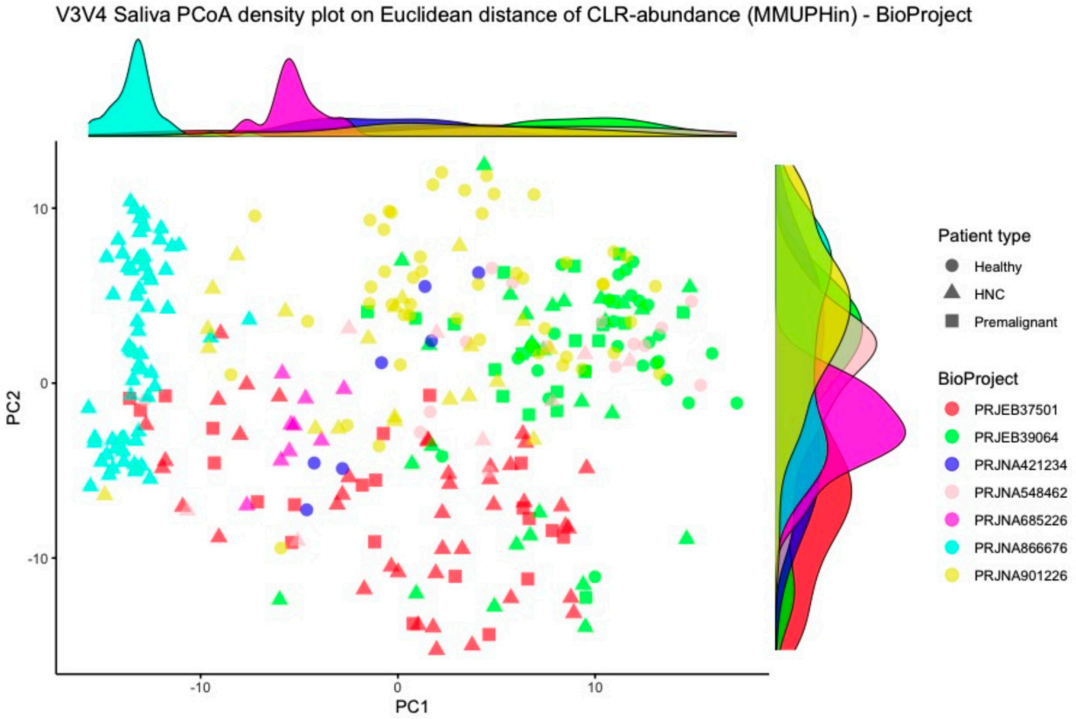
V3V4 Saliva PCoA density plot on Euclidean distance of CLR-abundance (MMUPHin) - BioProject
PC2
PC1
10
0
-10
-10
0
10
Patient type
Healthy
HNC
Premalignant
BioProject
PRJEB37501
PRJEB39064
PRJNA421234
PRJNA548462
PRJNA685226
PRJNA866676
PRJNA901226

(d)

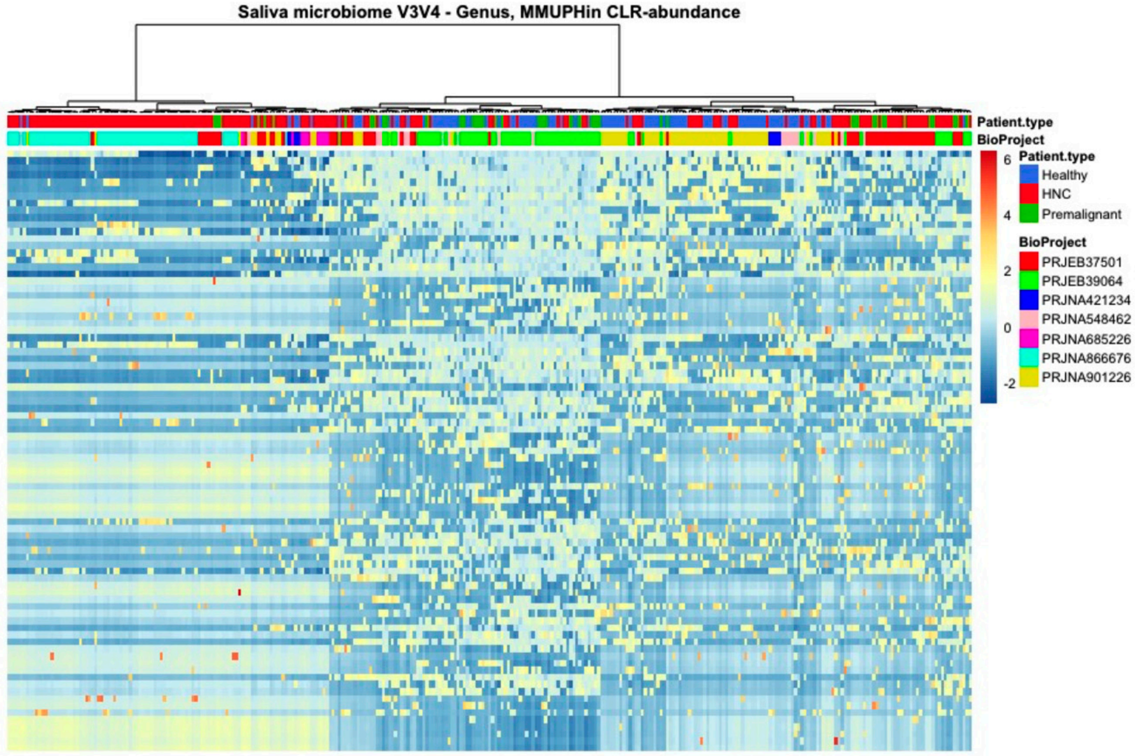
Saliva microbiome V3V4 - Genus, MMUPHin CLR-abundance
Patient.type
BioProject
6
4
2
0
-2
Patient.type
Healthy
HNC
Premalignant
BioProject
PRJEB37501
PRJEB39064
PRJNA421234
PRJNA548462
PRJNA685226
PRJNA866676
PRJNA901226

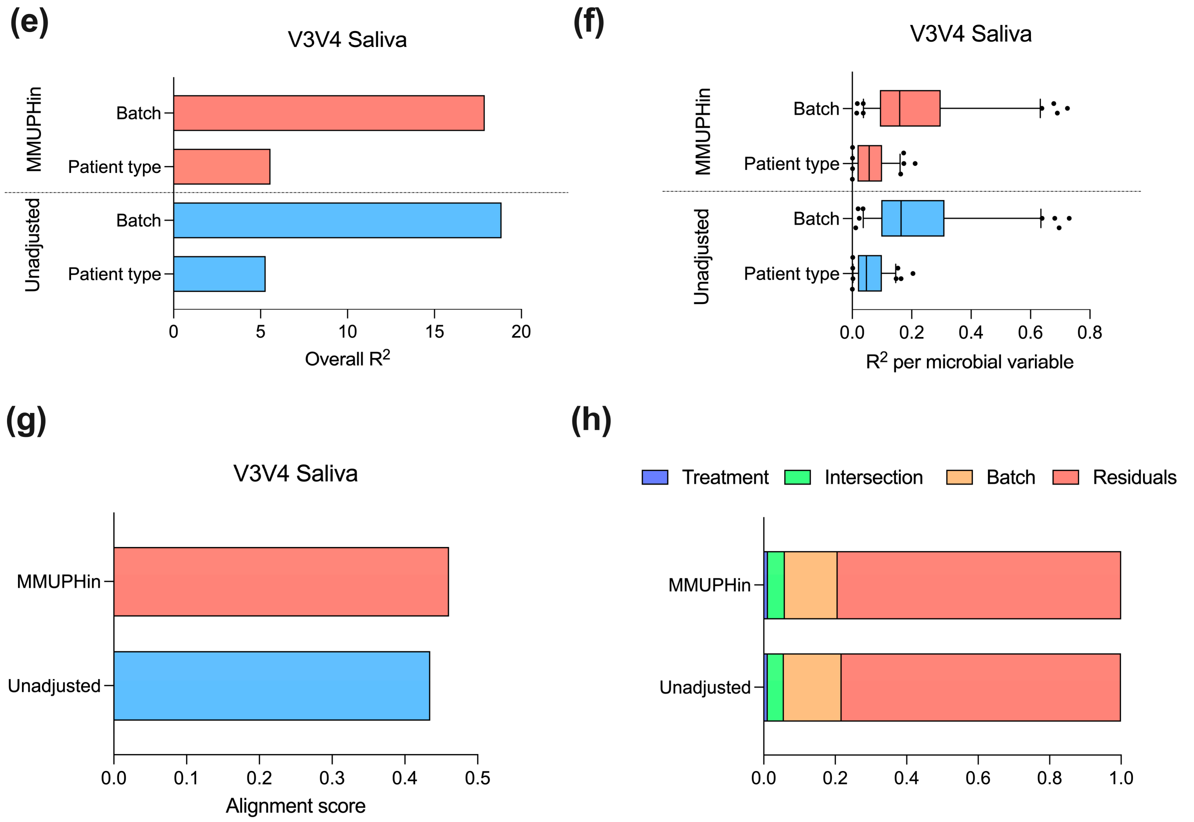

Figure S4: V3V4 saliva samples for unadjusted and after MMUPHin adjustment based on CLR-abundance. (a) PCA density plot and (b) heatmap for unadjusted CLR-abundance V3V4 of saliva samples. (c) PCA density plot and (d) heatmap for MMUPHin CLR-abundance of V3V4 saliva samples. Based on CoDA method, raw abundance counts were converted to CLR-abundance (offset = 0.5), and CLR-abundance were used for each plot. Study batches were represented by "BioProject". For heatmap, each column and row represent a unique sample and bacterial genera respectively, with OTUs clustered based on Euclidean distance and Ward linkage method. Heatmap was centred and scaled for visualisation. Evaluation of study batch effects using (e) Overall sum of R^2 values, (f) R^2 values for each microbial variable, (g) alignment score, and (h) Partial redundancy analysis (pRDA).

(a)

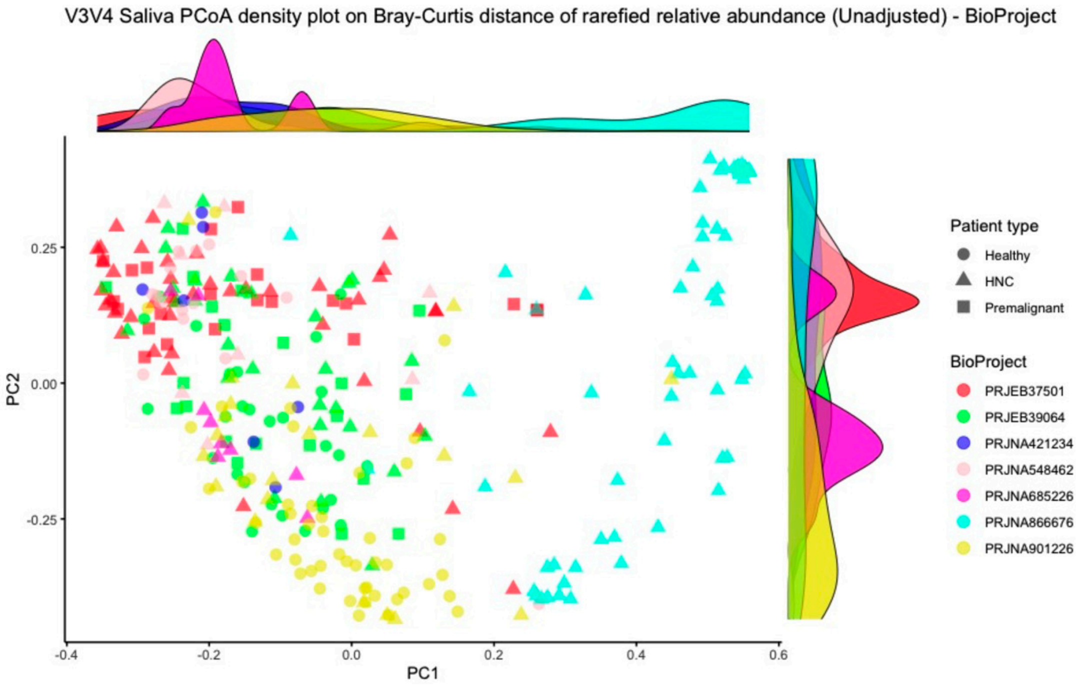
V3V4 Saliva PCoA density plot on Bray-Curtis distance of rarefied relative abundance (Unadjusted) - BioProject
PC1
PC2
-0.4
-0.2
0.0
0.2
0.4
0.6
0.25
0.00
-0.25
Patient type
Healthy
HNC
Premalignant
BioProject
PRJEB37501
PRJEB39064
PRJNA421234
PRJNA548462
PRJNA685226
PRJNA866676
PRJNA901226

(b)

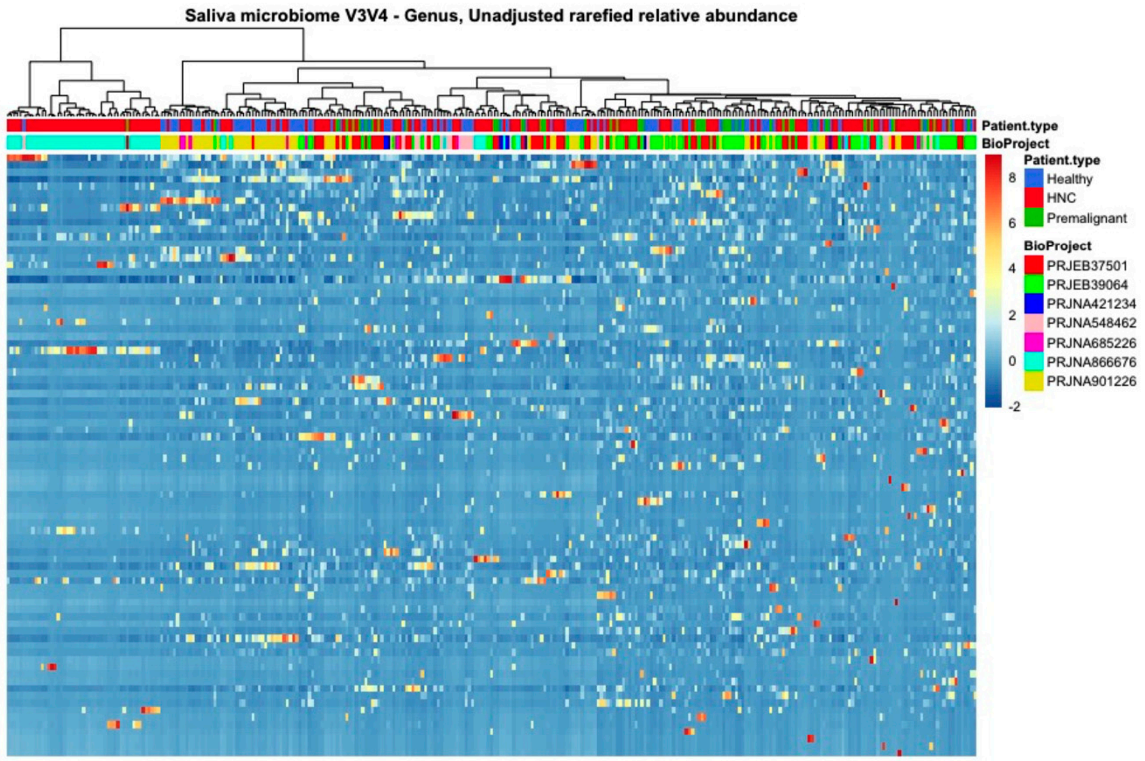
Saliva microbiome V3V4 - Genus, Unadjusted rarefied relative abundance
Patient.type
BioProject
8
6
4
2
0
-2
Patient.type
Healthy
HNC
Premalignant
BioProject
PRJEB37501
PRJEB39064
PRJNA421234
PRJNA548462
PRJNA685226
PRJNA866676
PRJNA901226

(c)

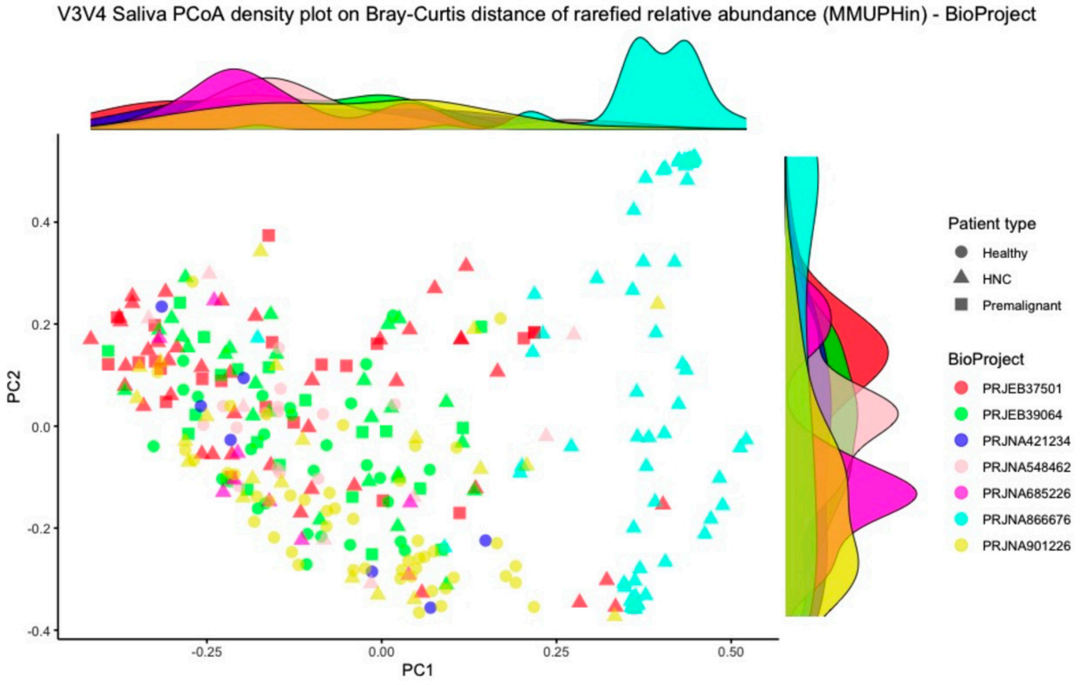
V3V4 Saliva PCoA density plot on Bray-Curtis distance of rarefied relative abundance (MMUPHin) - BioProject
PC2
PC1
0.4
0.2
0.0
-0.2
-0.4
-0.25
0.00
0.25
0.50
Patient type
Healthy
HNC
Premalignant
BioProject
PRJEB37501
PRJEB39064
PRJNA421234
PRJNA548462
PRJNA685226
PRJNA866676
PRJNA901226

(d)

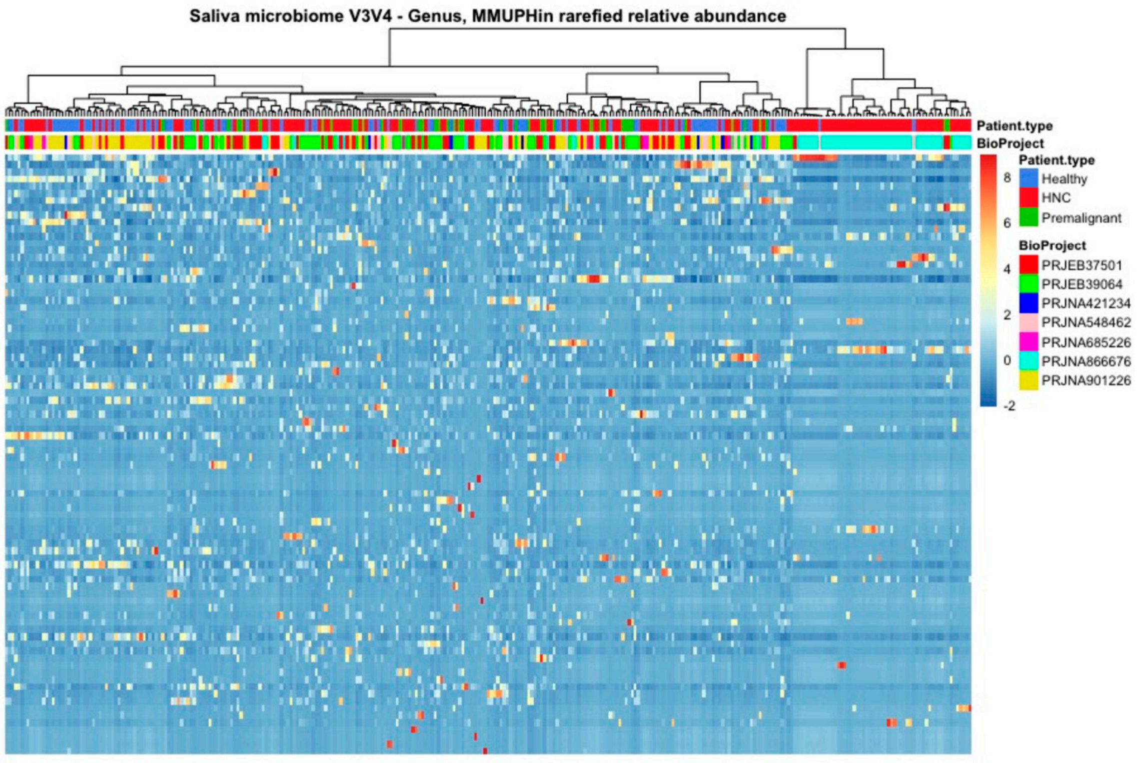
Saliva microbiome V3V4 - Genus, MMUPHin rarefied relative abundance
Patient.type
BioProject
8
6
4
2
0
-2
Patient.type
Healthy
HNC
Premalignant
BioProject
PRJEB37501
PRJEB39064
PRJNA421234
PRJNA548462
PRJNA685226
PRJNA866676
PRJNA901226

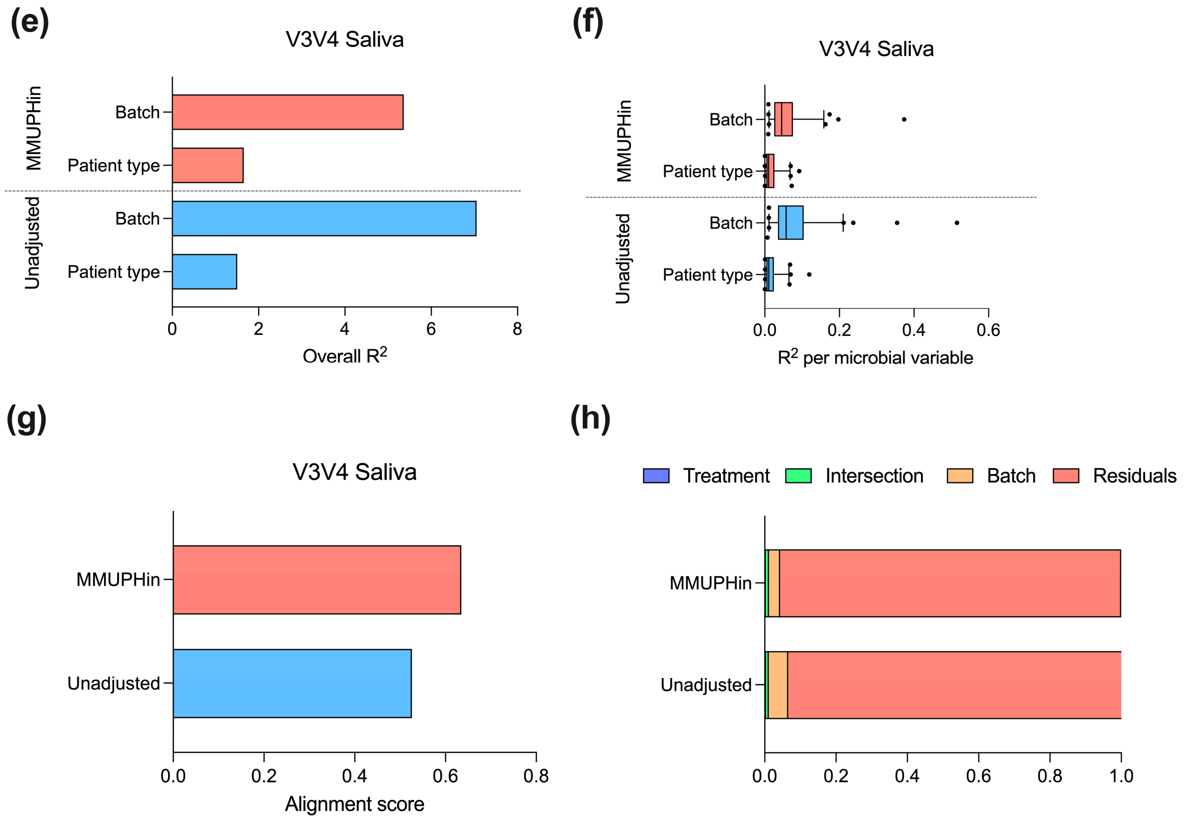

Figure S5: V3V4 saliva samples for unadjusted and after MMUPHin adjustment based on rarefied relative abundance. (a) PCA density plot and (b) heatmap for unadjusted rarefied relative abundance of V3V4 saliva samples. (c) PCA density plot and (d) heatmap for MMUPHin rarefied relative abundance of V3V4 saliva samples. Based on conventional analysis method, raw abundance counts were rarefied and converted to relative abundance for each plot. Study batches were represented by "BioProject". For heatmap, each column and row represent a unique sample and bacterial genera respectively, with OTUs clustered based on Euclidean distance and Ward linkage method. Heatmap was centred and scaled for visualisation. Evaluation of study batch effects using (e) Overall sum of R^2 values, (f) R^2 values for each microbial variable, (g) alignment score, and (h) Partial redundancy analysis (pRDA).

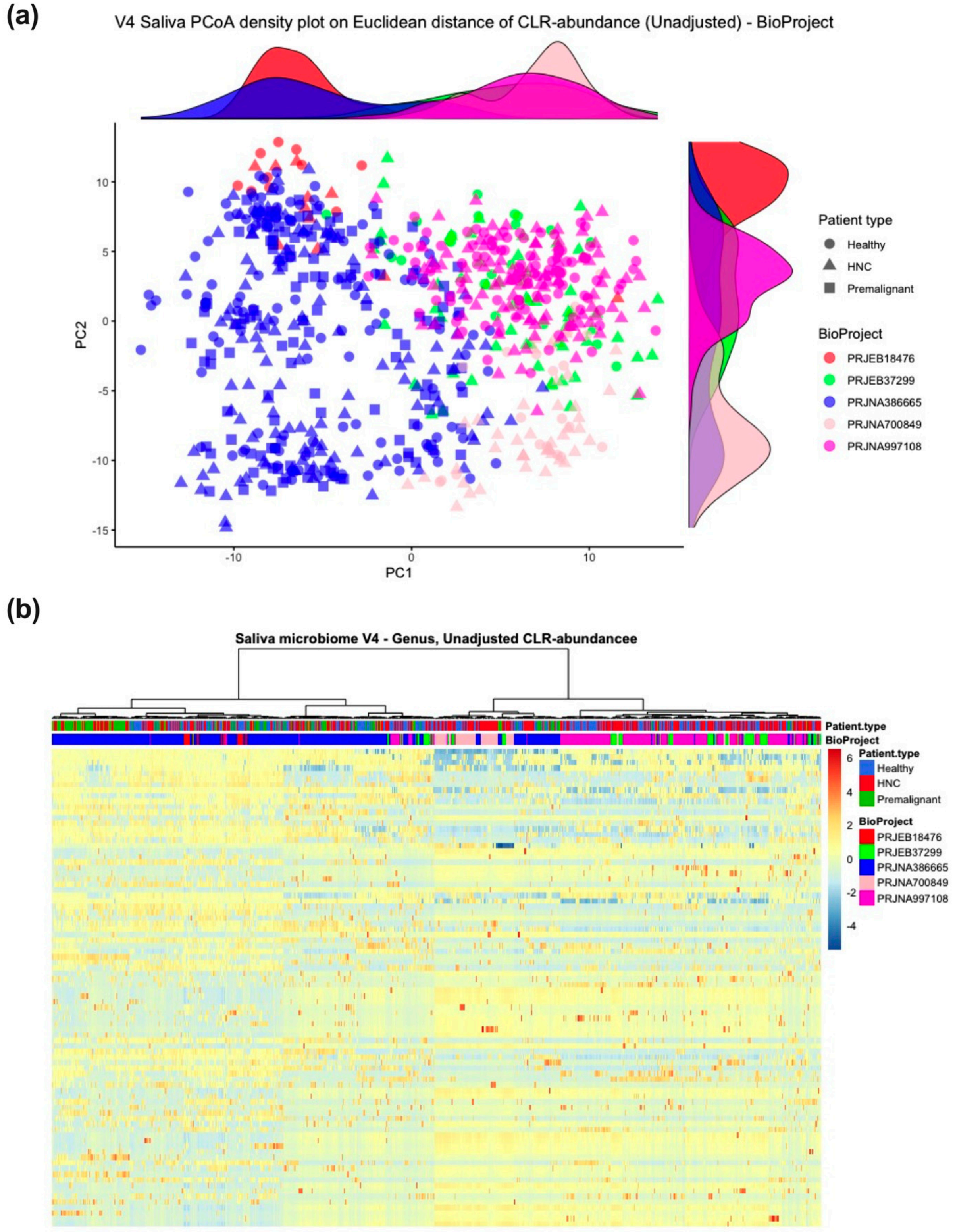
(a)
V4 Saliva PCoA density plot on Euclidean distance of CLR-abundance (Unadjusted) - BioProject
PC1
PC2
10
5
0
-5
-10
-15
-10
0
10
Patient type
Healthy
HNC
Premalignant
BioProject
PRJEB18476
PRJEB37299
PRJNA386665
PRJNA700849
PRJNA997108
(b)
Saliva microbiome V4 - Genus, Unadjusted CLR-abundancee
Patient.type
BioProject
6
4
2
0
-2
-4
Patient.type
Healthy
HNC
Premalignant
BioProject
PRJEB18476
PRJEB37299
PRJNA386665
PRJNA700849
PRJNA997108

(c)

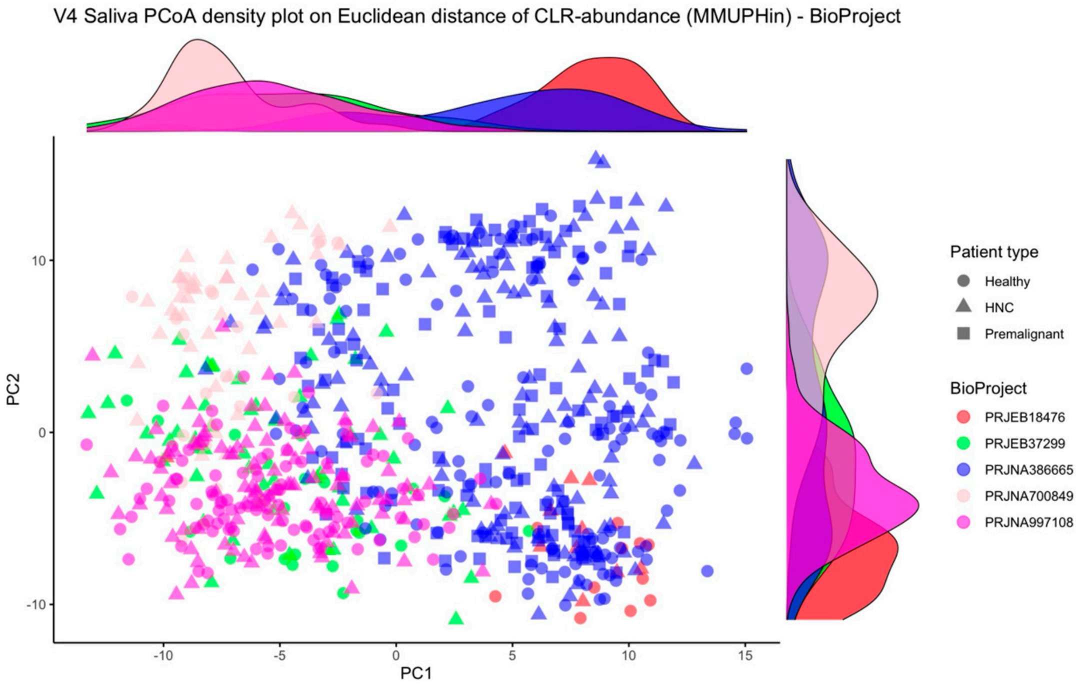

V4 Saliva PCoA density plot on Euclidean distance of CLR-abundance (MMUPHin) - BioProject
PC1
PC2
Patient type
Healthy
HNC
Premalignant
BioProject
PRJEB18476
PRJEB37299
PRJNA386665
PRJNA700849
PRJNA997108

(d)

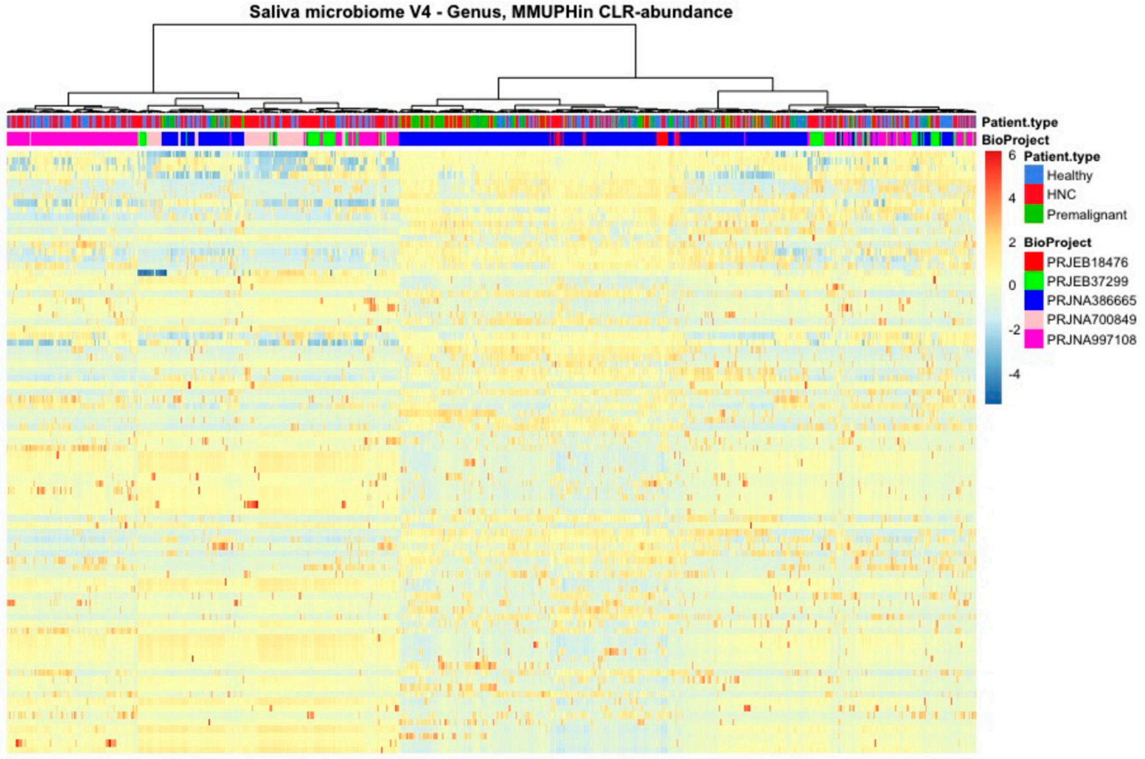

Saliva microbiome V4 - Genus, MMUPHin CLR-abundance
Patient.type
BioProject
Patient.type
Healthy
HNC
Premalignant
BioProject
PRJEB18476
PRJEB37299
PRJNA386665
PRJNA700849
PRJNA997108

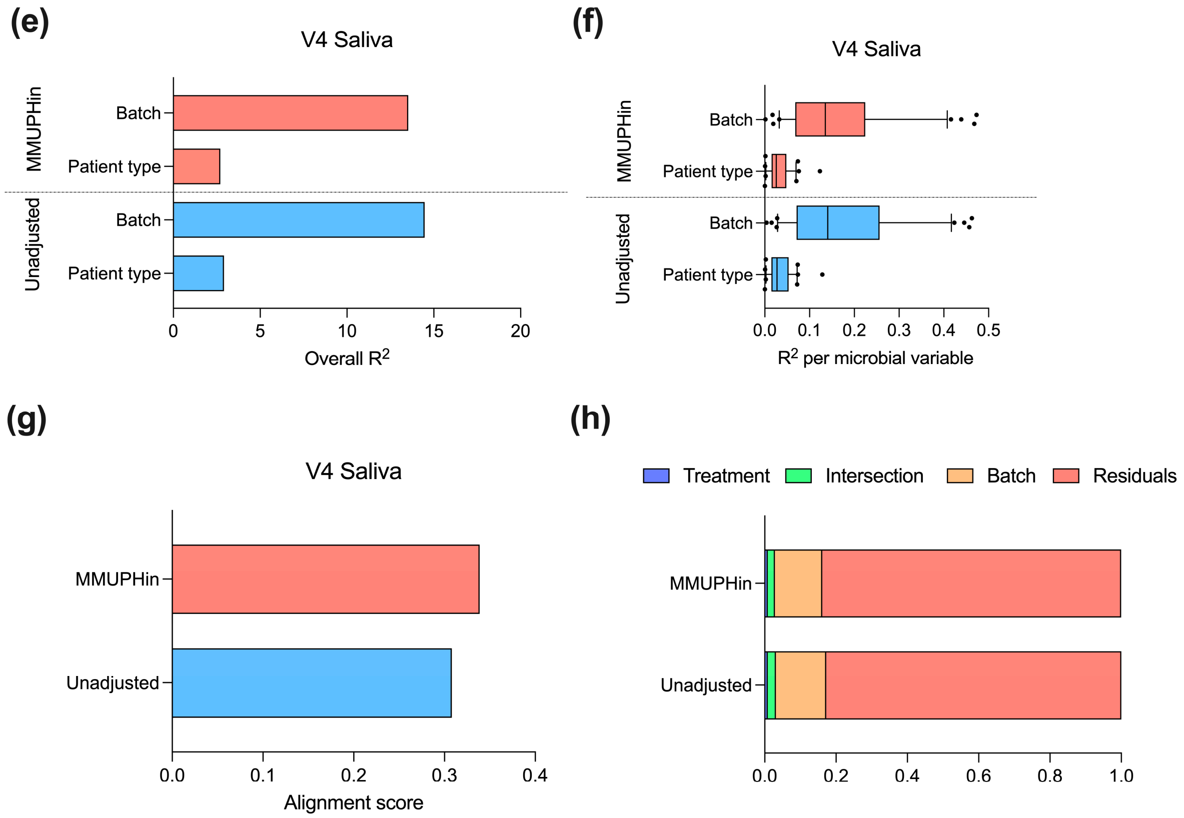

Figure S6: V4 saliva samples for unadjusted and after MMUPHin adjustment based on CLR-abundance. (a) PCA density plot and (b) heatmap for unadjusted CLR-abundance V4 of saliva samples. (c) PCA density plot and (d) heatmap for MMUPHin CLR-abundance of V4 saliva samples. Based on CoDA method, raw abundance counts were converted to CLR-abundance (offset = 0.5), and CLR-abundance were used for each plot. Study batches were represented by "BioProject". For heatmap, each column and row represent a unique sample and bacterial genera respectively, with OTUs clustered based on Euclidean distance and Ward linkage method. Heatmap was centred and scaled for visualisation. Evaluation of study batch effects using (e) Overall sum of R^2 values, (f) R^2 values for each microbial variable, (g) alignment score, and (h) Partial redundancy analysis (pRDA).

(a)

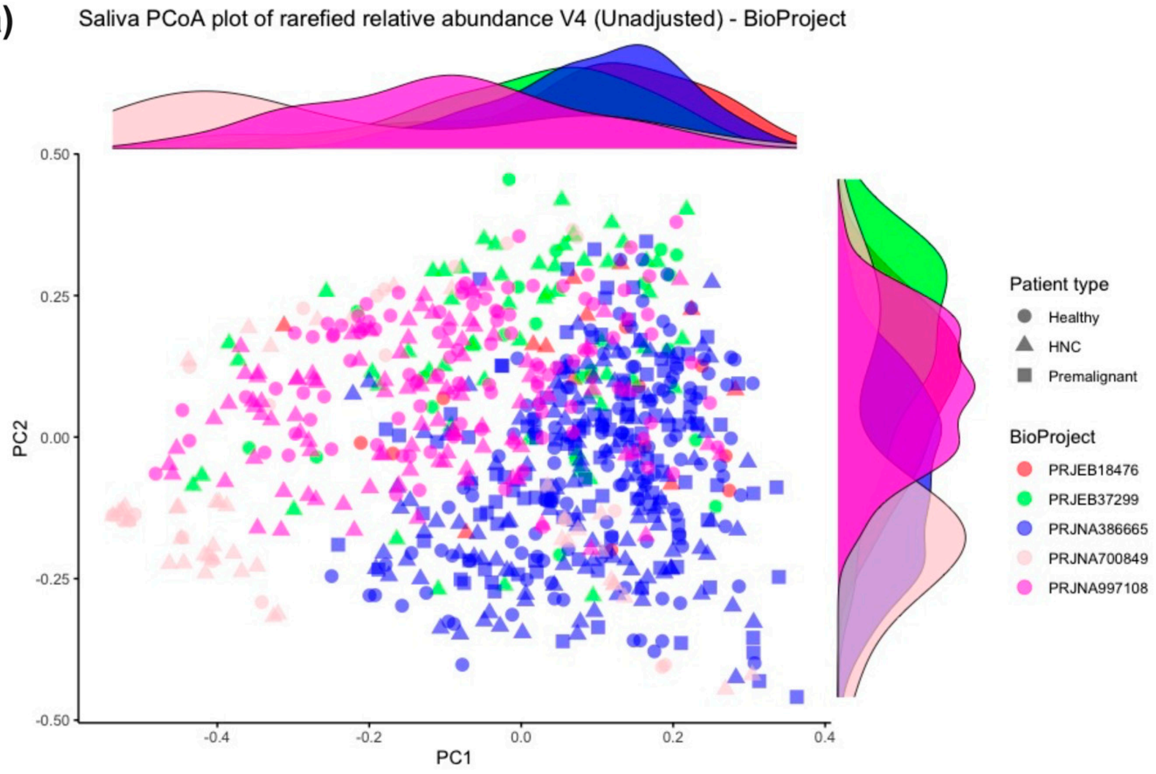
Saliva PCoA plot of rarefied relative abundance V4 (Unadjusted) - BioProject
PC1
PC2
0.50
0.25
0.00
-0.25
-0.50
-0.4
-0.2
0.0
0.2
0.4
Patient type
Healthy
HNC
Premalignant
BioProject
PRJEB18476
PRJEB37299
PRJNA386665
PRJNA700849
PRJNA997108

(b)

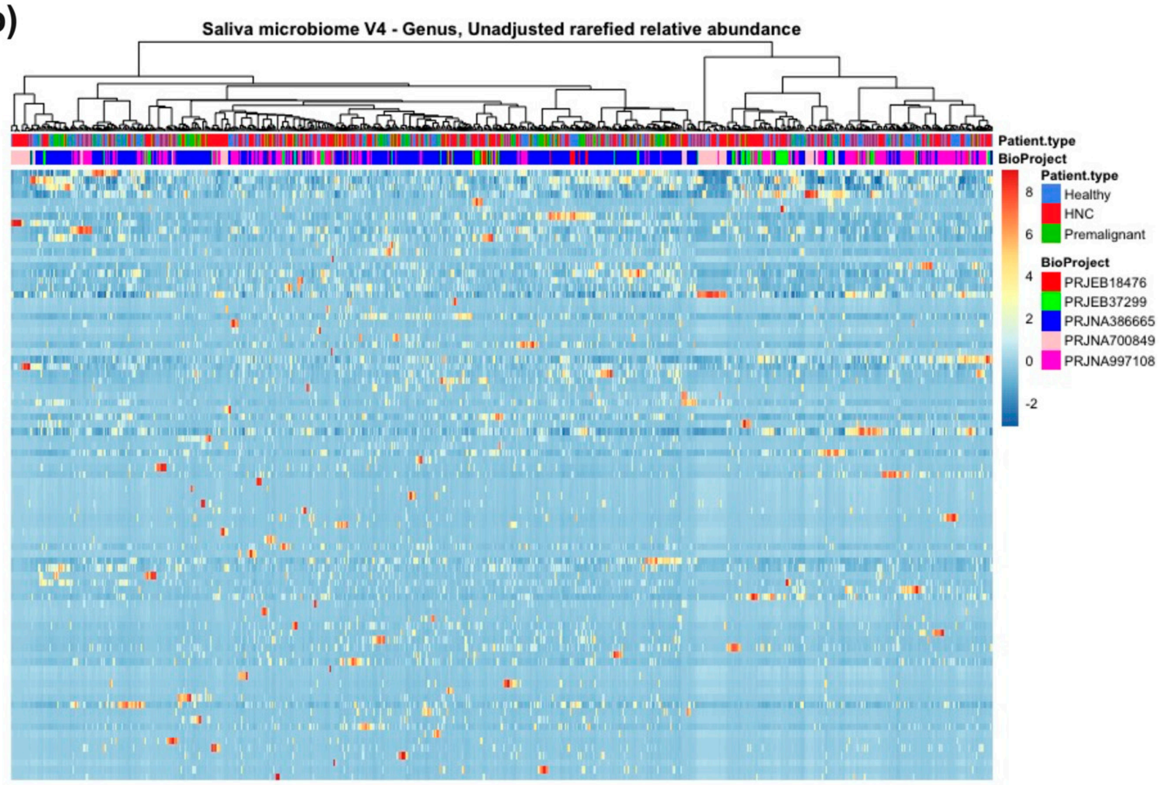
Saliva microbiome V4 - Genus, Unadjusted rarefied relative abundance
Patient.type
BioProject
8
6
4
2
0
-2
Patient.type
Healthy
HNC
Premalignant
BioProject
PRJEB18476
PRJEB37299
PRJNA386665
PRJNA700849
PRJNA997108

(c)

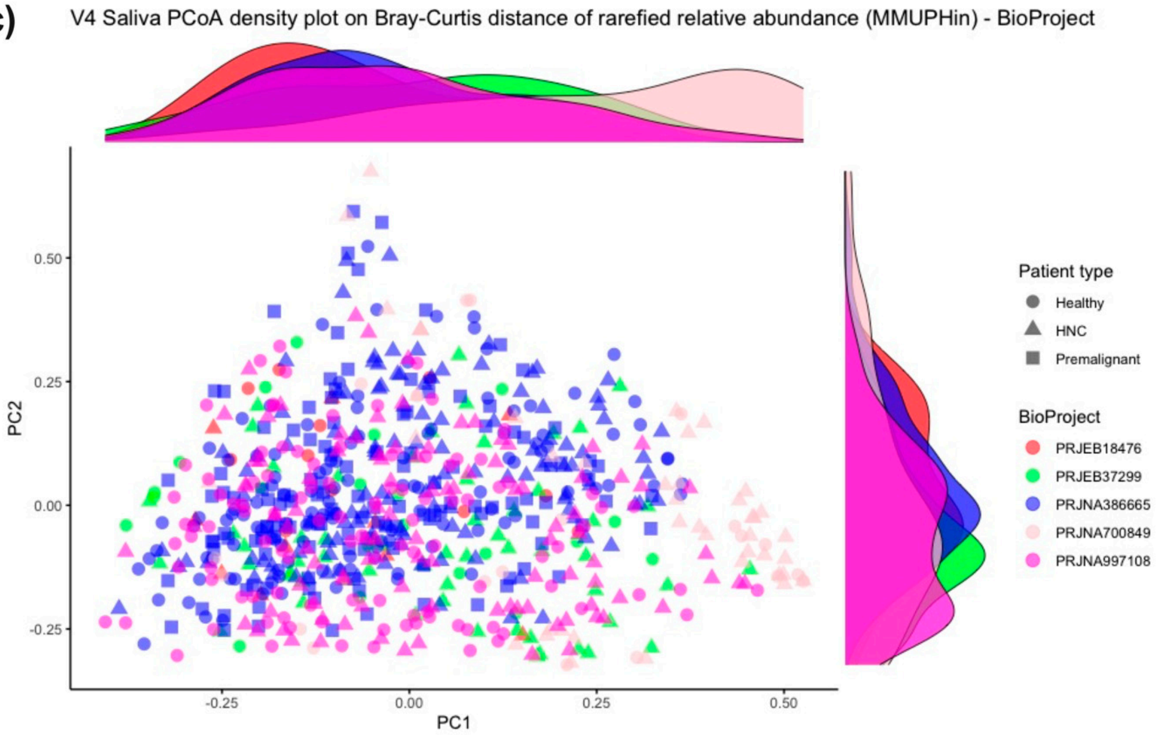
V4 Saliva PCoA density plot on Bray-Curtis distance of rarefied relative abundance (MMUPHin) - BioProject
PC2
0.50
0.25
0.00
-0.25
PC1
-0.25
0.00
0.25
0.50
Patient type
Healthy
HNC
Premalignant
BioProject
PRJEB18476
PRJEB37299
PRJNA386665
PRJNA700849
PRJNA997108

(d)

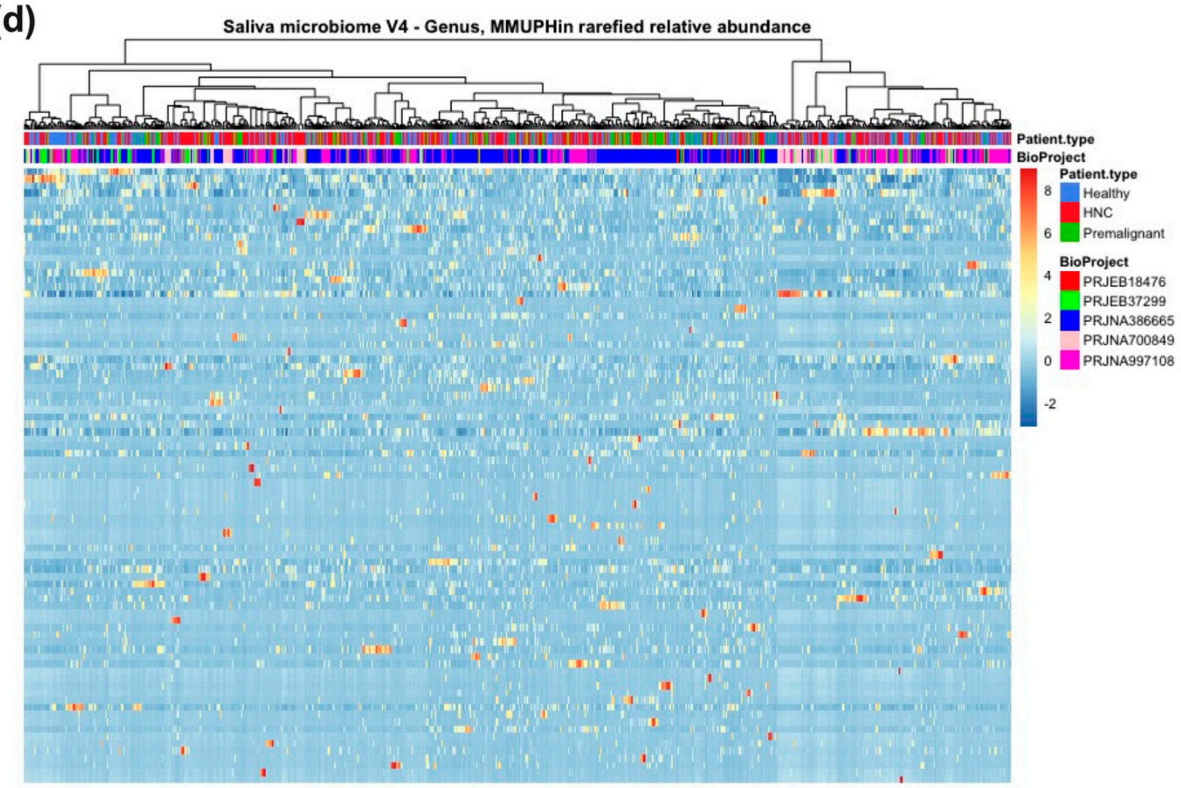
Saliva microbiome V4 - Genus, MMUPHin rarefied relative abundance
Patient.type
BioProject
8
6
4
2
0
-2
Patient.type
Healthy
HNC
Premalignant
BioProject
PRJEB18476
PRJEB37299
PRJNA386665
PRJNA700849
PRJNA997108

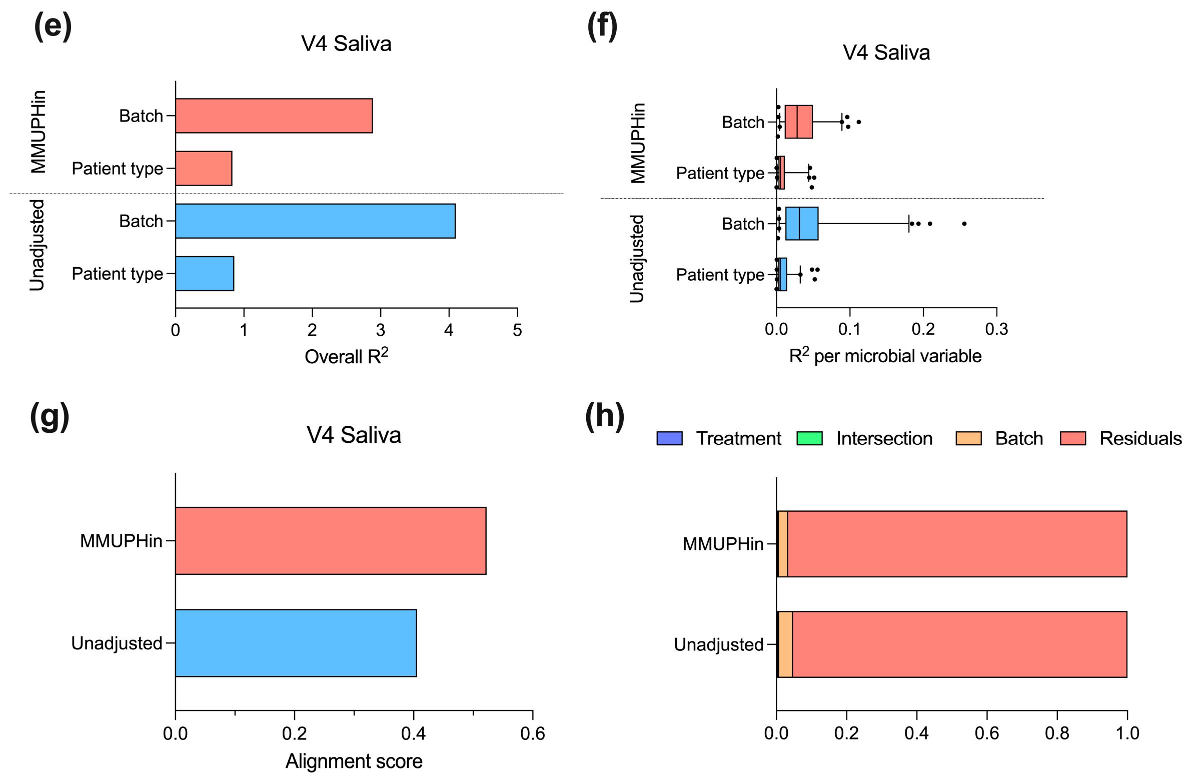

Figure S7: V4 saliva samples for unadjusted and after MMUPHin adjustment based on rarefied relative abundance. (a) PCA density plot and (b) heatmap for unadjusted rarefied relative abundance of V4 saliva samples. (c) PCA density plot and (d) heatmap for MMUPHin rarefied relative abundance of V4 saliva samples. Based on conventional analysis method, raw abundance counts were rarefied and converted to relative abundance for each plot. Study batches were represented by "BioProject". For heatmap, each column and row represent a unique sample and bacterial genera respectively, with OTUs clustered based on Euclidean distance and Ward linkage method. Heatmap was centred and scaled for visualisation. Evaluation of study batch effects using (e) Overall sum of R^2 values, (f) R^2 values for each microbial variable, (g) alignment score, and (h) Partial redundancy analysis (pRDA).

(a)

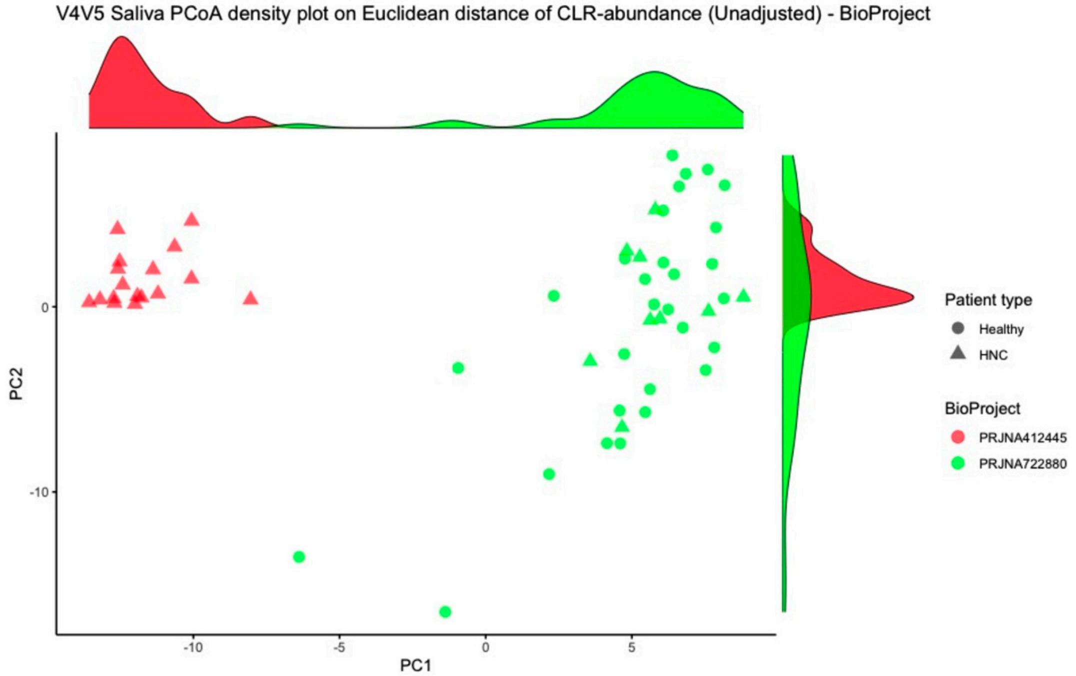

V4V5 Saliva PCoA density plot on Euclidean distance of CLR-abundance (Unadjusted) - BioProject
PC1
PC2
-10
-5
0
5
Patient type
Healthy
HNC
BioProject
PRJNA412445
PRJNA722880

(b)

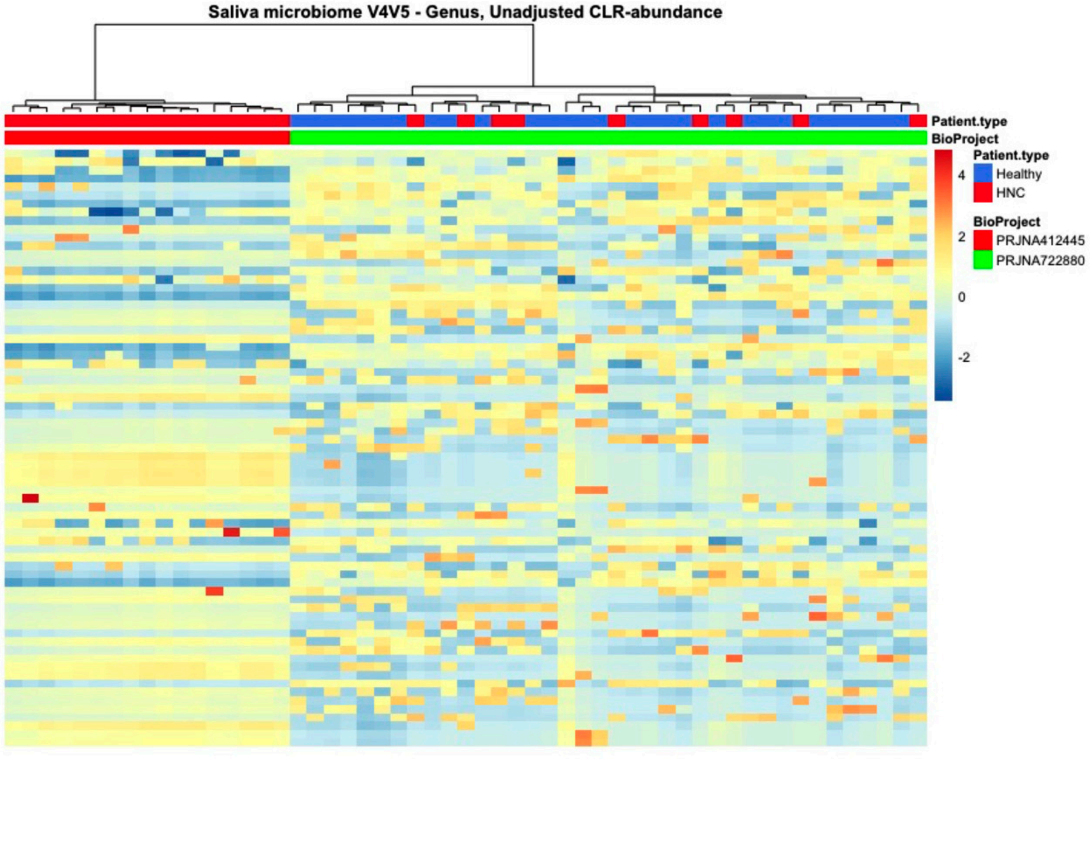

Saliva microbiome V4V5 - Genus, Unadjusted CLR-abundance
Patient.type
BioProject
Patient.type
Healthy
HNC
BioProject
PRJNA412445
PRJNA722880
4
2
0
-2

(c)

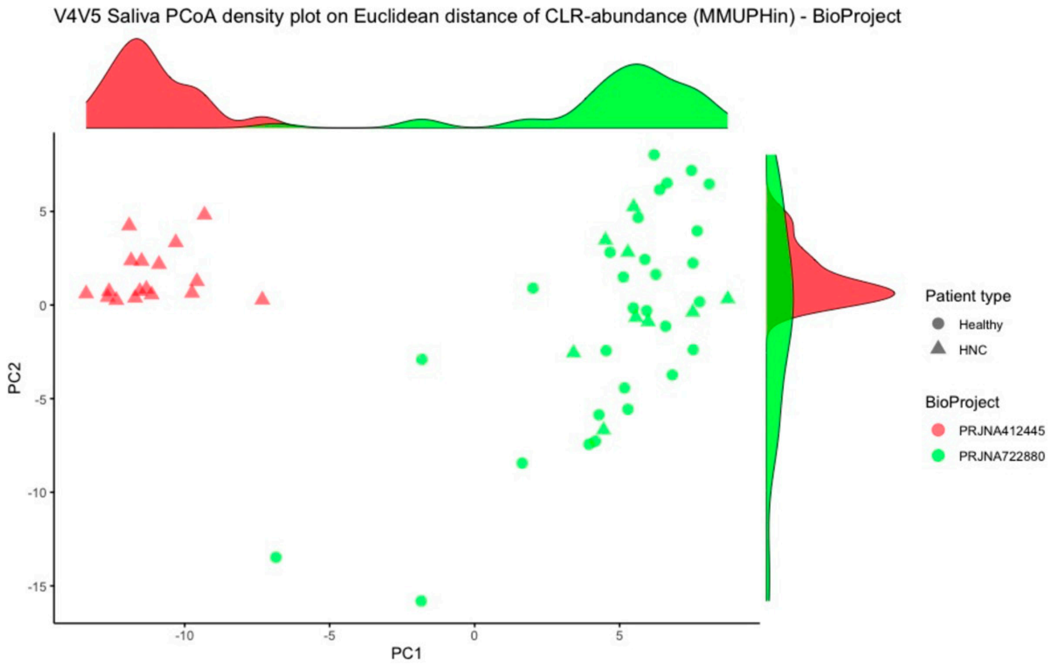
V4V5 Saliva PCoA density plot on Euclidean distance of CLR-abundance (MMUPHin) - BioProject
PC2
PC1
5
0
-5
-10
-15
-10
-5
0
5
Patient type
Healthy
HNC
BioProject
PRJNA412445
PRJNA722880

(d)

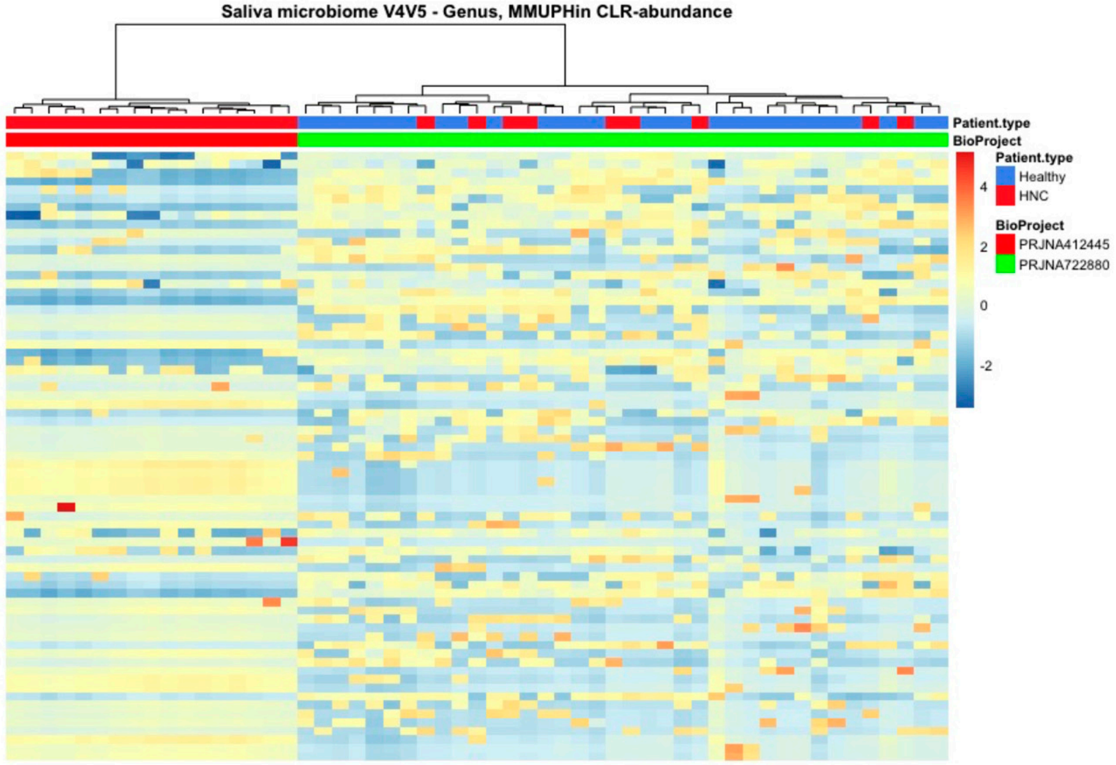
Saliva microbiome V4V5 - Genus, MMUPHin CLR-abundance
Patient.type
BioProject
Patient.type
Healthy
HNC
BioProject
PRJNA412445
PRJNA722880
4
2
0
-2

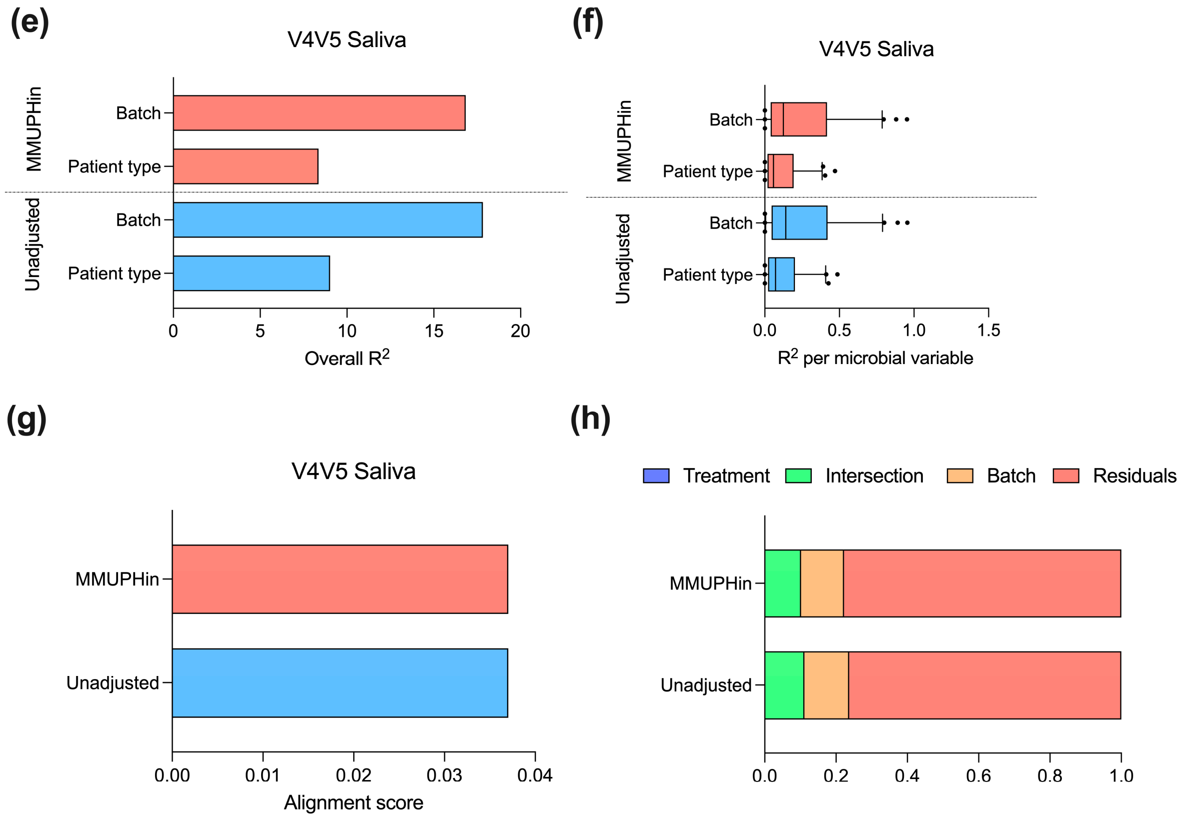

Figure S8: V4V5 saliva samples for unadjusted and after MMUPHin adjustment based on CLR-abundance. (a) PCA density plot and (b) heatmap for unadjusted CLR-abundance of V4V5 saliva samples. (c) PCA density plot and (d) heatmap for MMUPHin CLR-abundance of V4V5 saliva samples. Based on conventional analysis method, raw abundance counts were converted to CLR-abundance for each plot. Study batches were represented by "BioProject". For heatmap, each column and row represent a unique sample and bacterial genera respectively, with OTUs clustered based on Euclidean distance and Ward linkage method. Heatmap was centred and scaled for visualisation. Evaluation of study batch effects using (e) Overall sum of R^2 values, (f) R^2 values for each microbial variable, (g) alignment score, and (h) Partial redundancy analysis (pRDA).

(a)

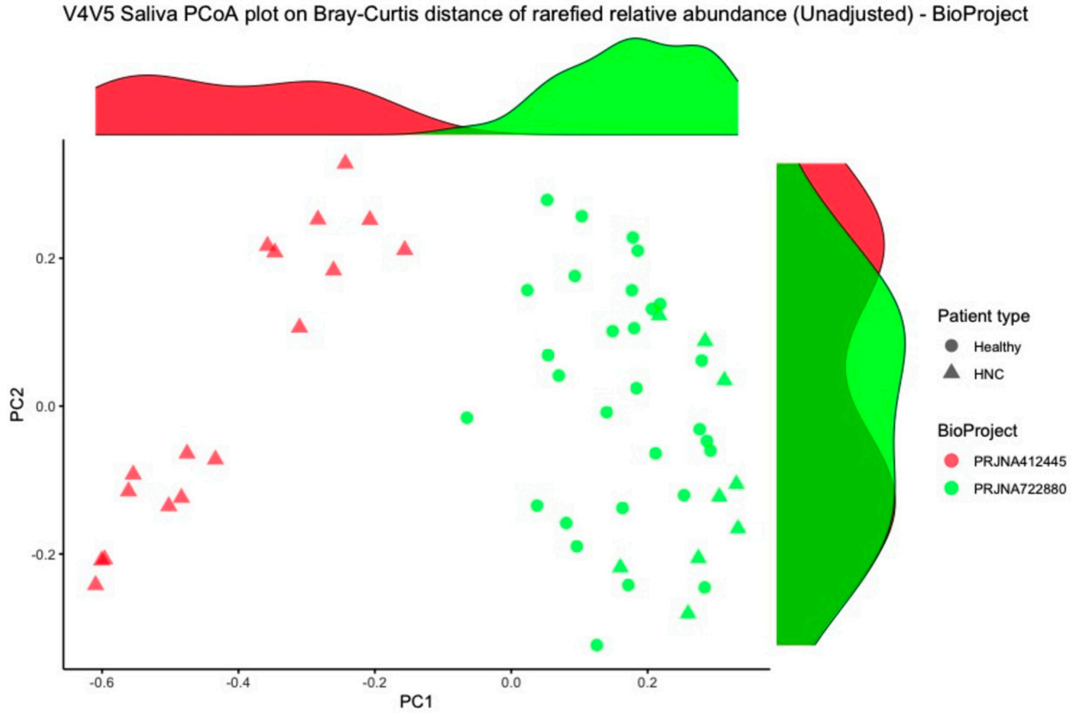
V4V5 Saliva PCoA plot on Bray-Curtis distance of rarefied relative abundance (Unadjusted) - BioProject
PC1
PC2
Patient type
Healthy
HNC
BioProject
PRJNA412445
PRJNA722880

(b)

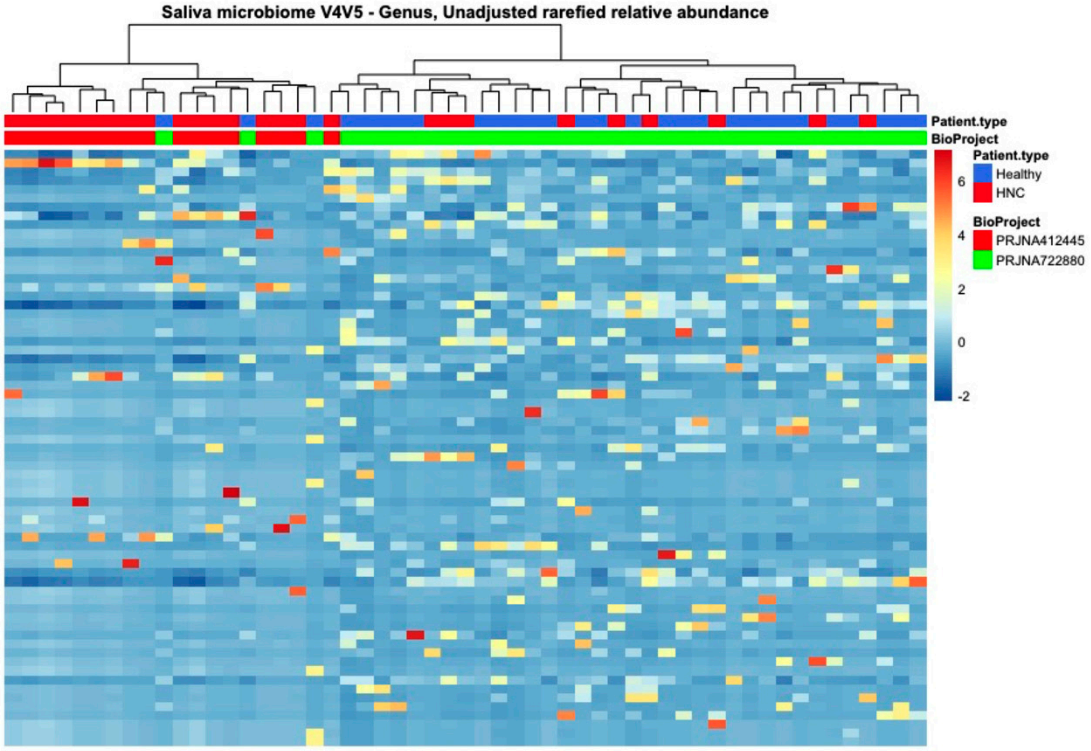
Saliva microbiome V4V5 - Genus, Unadjusted rarefied relative abundance
Patient.type
BioProject
Patient.type
Healthy
HNC
BioProject
PRJNA412445
PRJNA722880

(c)

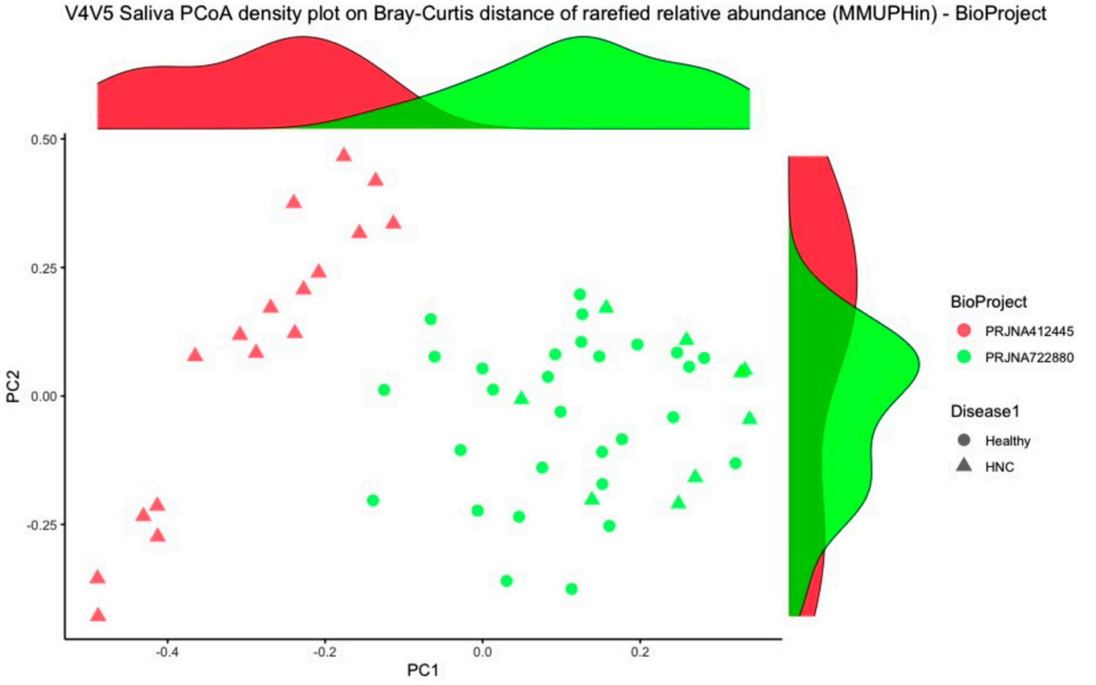

V4V5 Saliva PCoA density plot on Bray-Curtis distance of rarefied relative abundance (MMUPHin) - BioProject
0.50
0.25
0.00
-0.25
PC2
-0.4
-0.2
0.0
0.2
PC1
BioProject
PRJNA412445
PRJNA722880
Disease1
Healthy
HNC

(d)

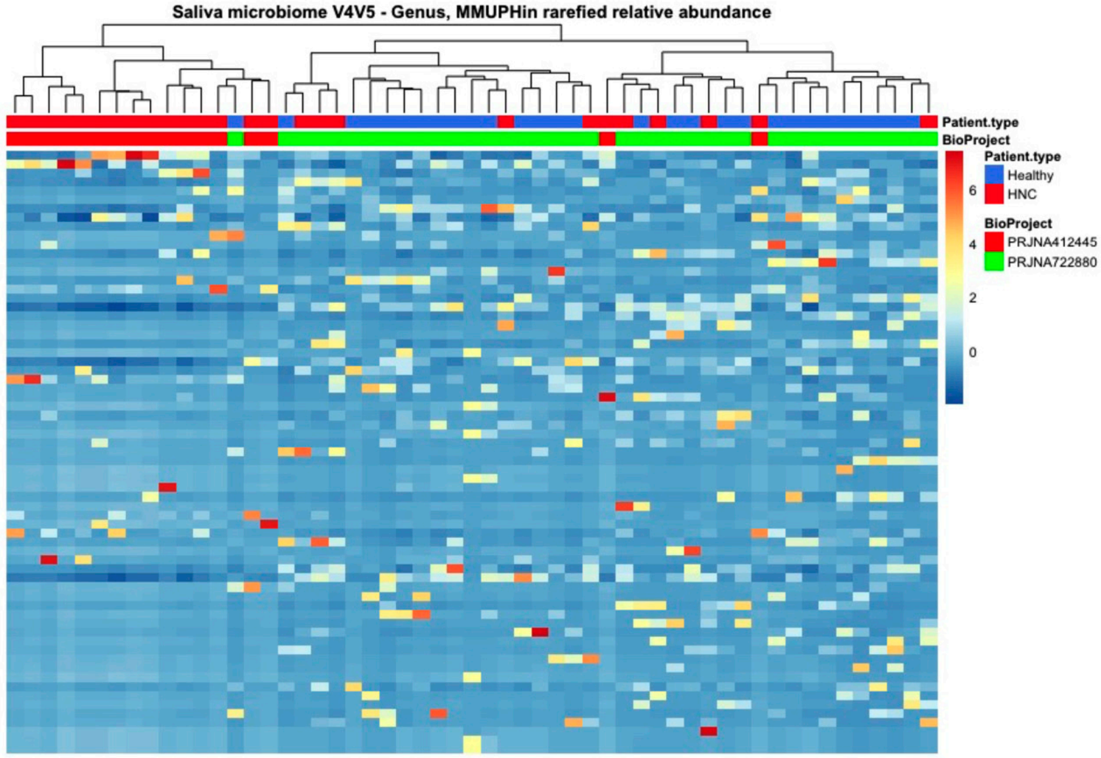

Saliva microbiome V4V5 - Genus, MMUPHin rarefied relative abundance
Patient.type
BioProject
6
4
2
0
Patient.type
Healthy
HNC
BioProject
PRJNA412445
PRJNA722880

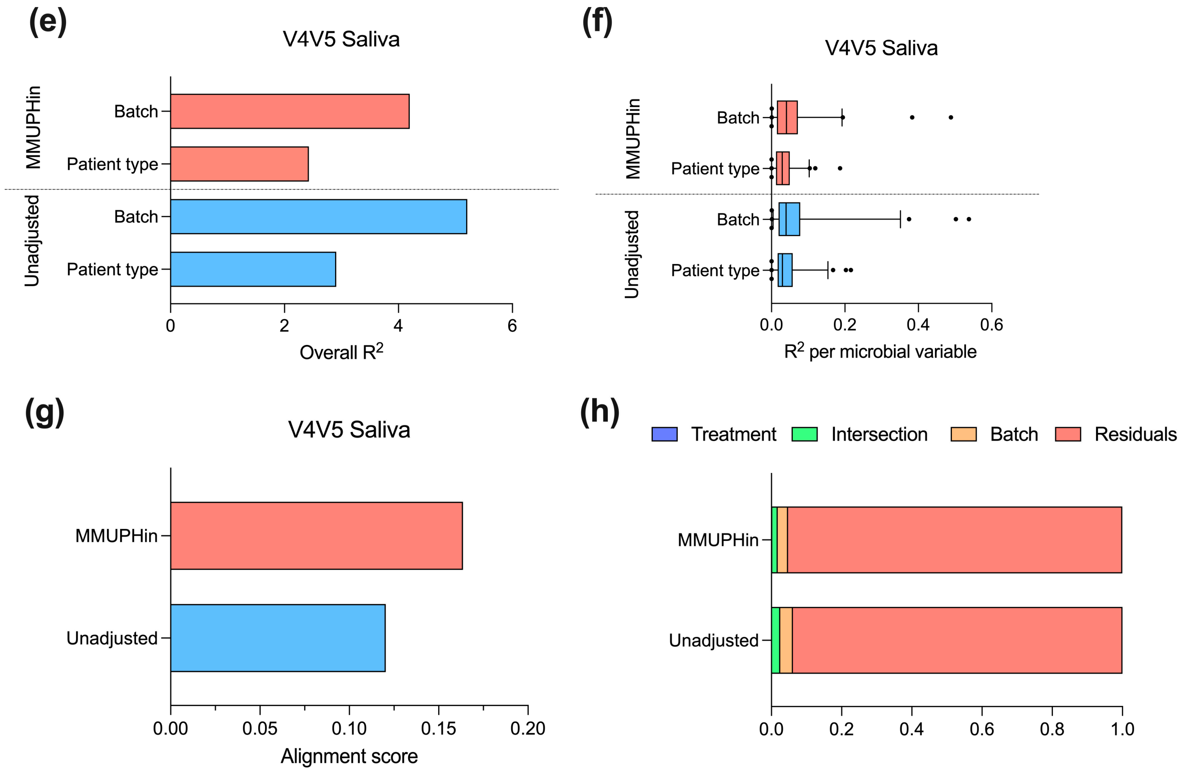

Figure S9: V4V5 saliva samples for unadjusted and after MMUPHin adjustment based on rarefied relative abundance. (a) PCA density plot and (b) heatmap for unadjusted rarefied relative abundance of V4V5 saliva samples. (c) PCA density plot and (d) heatmap for MMUPHin rarefied relative abundance of V4V5 saliva samples. Based on conventional analysis method, raw abundance counts were rarefied and converted to relative abundance for each plot. Study batches were represented by "BioProject". For heatmap, each column and row represent a unique sample and bacterial genera respectively, with OTUs clustered based on Euclidean distance and Ward linkage method. Heatmap was centred and scaled for visualisation. Evaluation of study batch effects using (e) Overall sum of R^2 values, (f) R^2 values for each microbial variable, (g) alignment score, and (h) Partial redundancy analysis (pRDA).

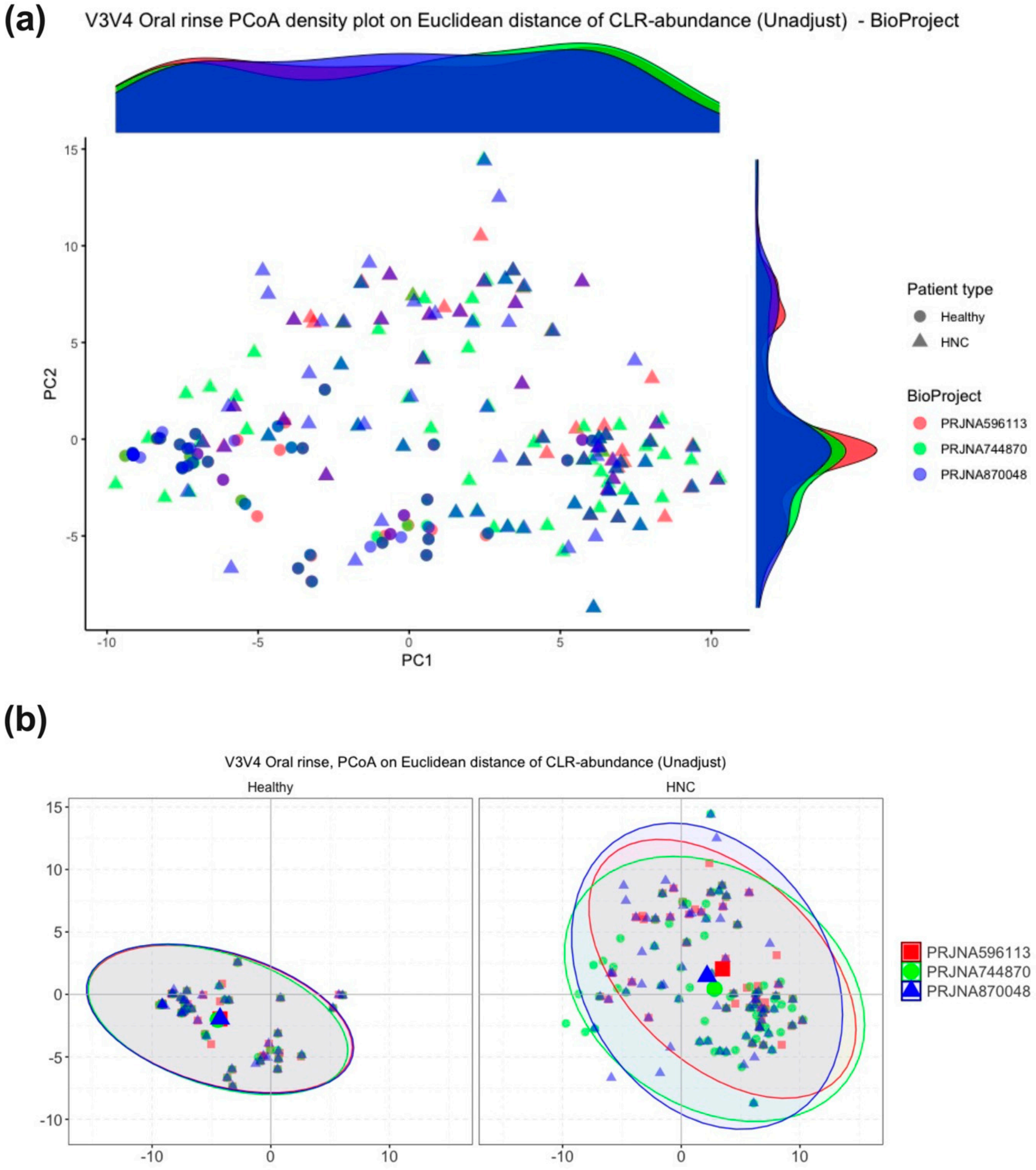
(a)
V3V4 Oral rinse PCoA density plot on Euclidean distance of CLR-abundance (Unadjust) - BioProject
PC1
PC2
Patient type
Healthy
HNC
BioProject
PRJNA596113
PRJNA744870
PRJNA870048
(b)
V3V4 Oral rinse, PCoA on Euclidean distance of CLR-abundance (Unadjust)
Healthy
HNC
PRJNA596113
PRJNA744870
PRJNA870048

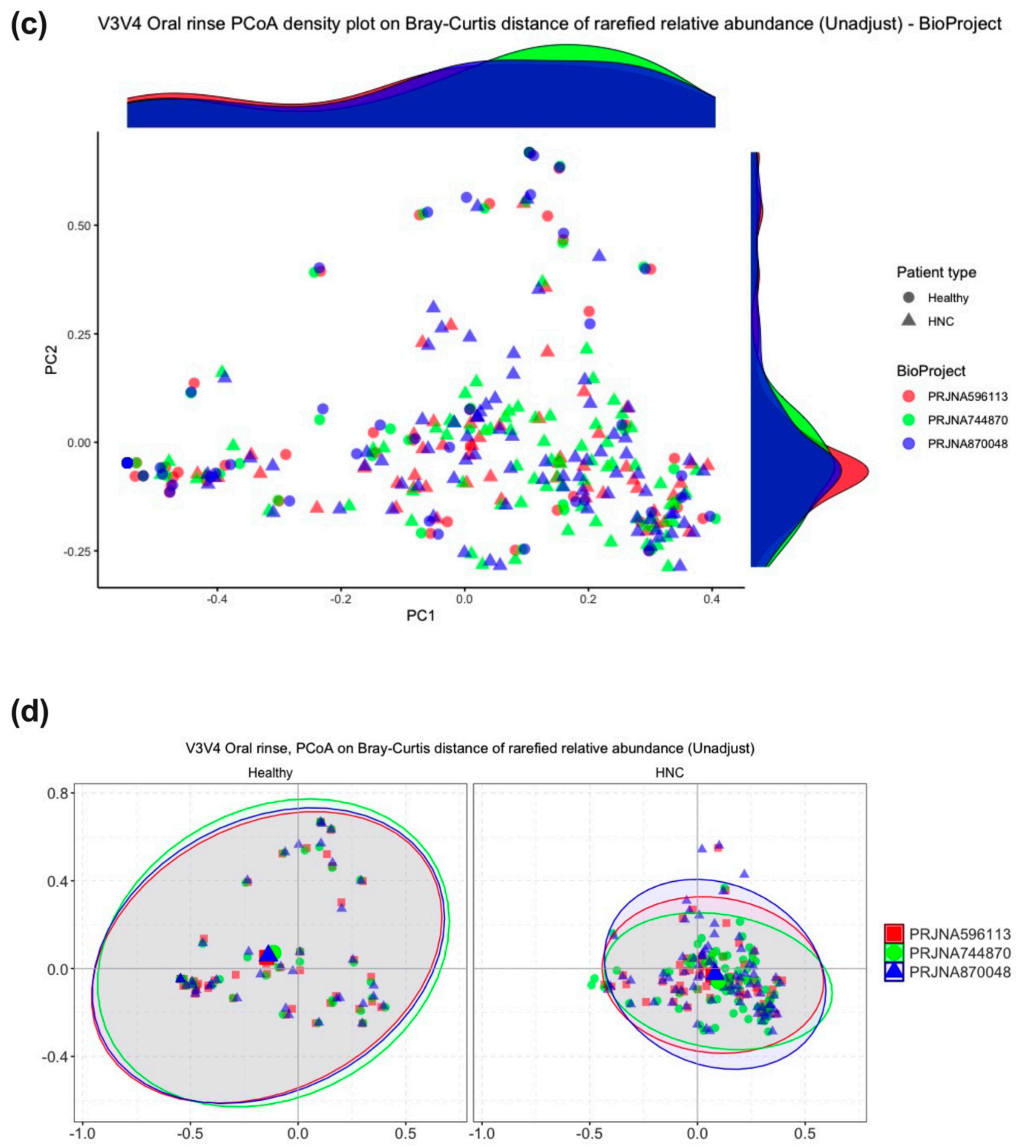

Figure S10: PCoA density plot on for V3V4 unadjusted oral rinse samples (HNC = 206, Healthy = 130). (a) PCoA density plot and (b) PCoA plot facet by patient type based on Euclidean distance of CLR-abundance. (c) PCoA density plot and (d) PCoA plot facet by patient type based on Bray-Curtis distance of rarefied relative abundance.

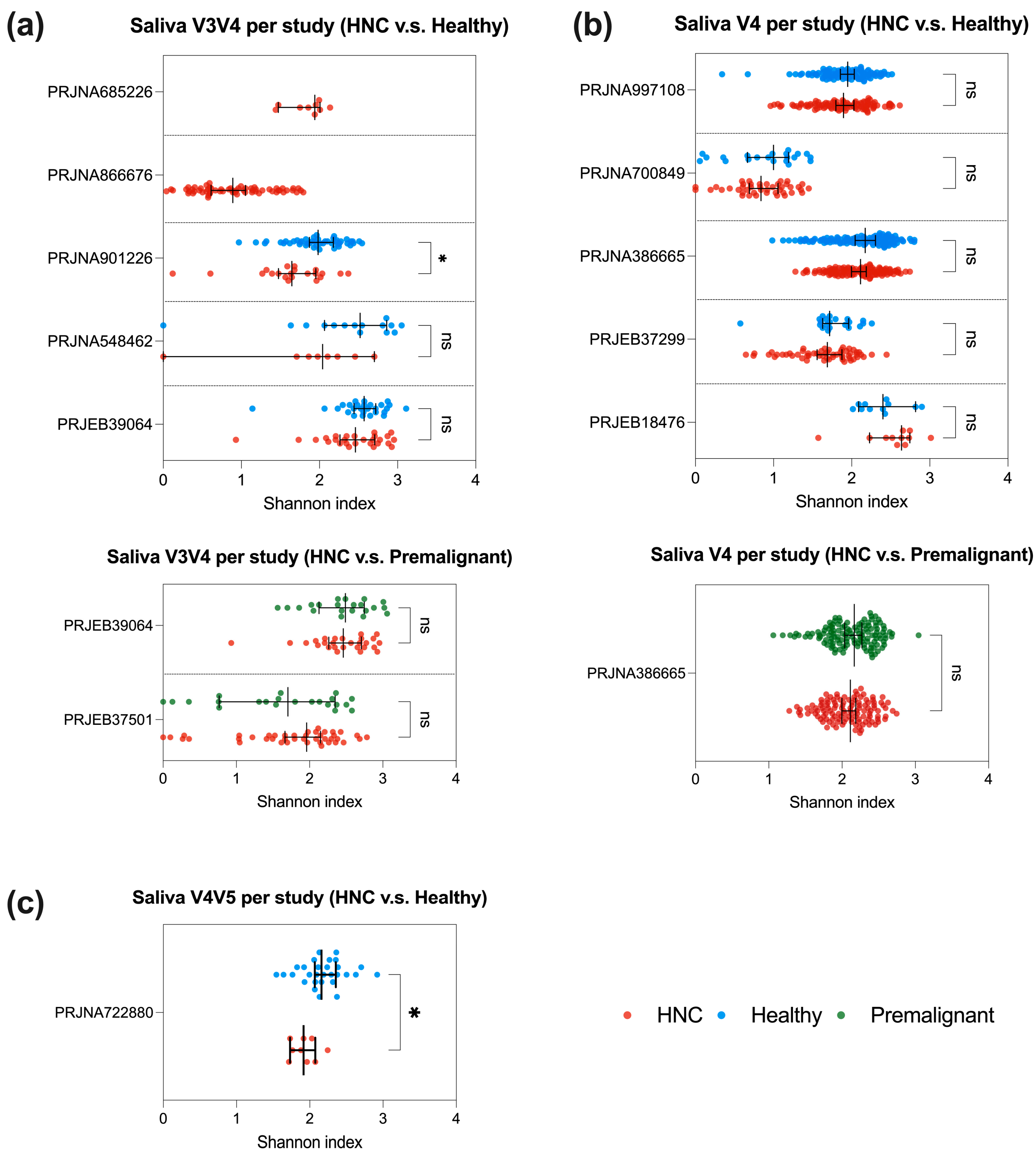

Figure S11. Comparison of alpha-diversity per study for HNC, premalignant and healthy saliva samples. Shannon diversity was measured for (A) V3V4, (B) V4, and (C) V4V5 saliva samples using rarefied abundance at the genera level. HNC saliva samples were compared to premalignant or healthy samples using Mann-Whitney test, with median ± 95% CI presented. For V3V4 samples, PRJNA685226 and PRJNA866676 only contained HNC samples. ****$p < 0.0001$, ***$p < 0.001$, **$p < 0.01$, *$p < 0.05$, ns - not significant.

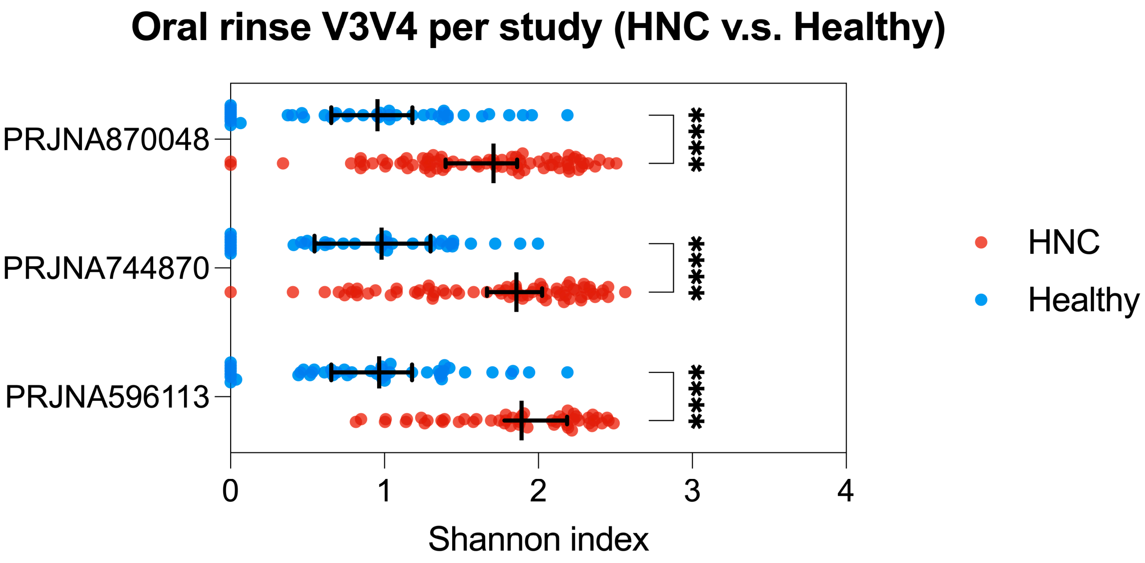

Figure S12. Comparison of alpha-diversity per study for HNC, premalignant and healthy oral rinse samples. Shannon diversity was measured for V3V4 oral rinse samples using rarefied abundance at the genera level. HNC saliva samples were compared to healthy samples using Mann-Whitney test, with median ± 95% CI presented. ****$p < 0.0001$, ***$p < 0.001$, **$p < 0.01$, *$p < 0.05$, ns – not significant.

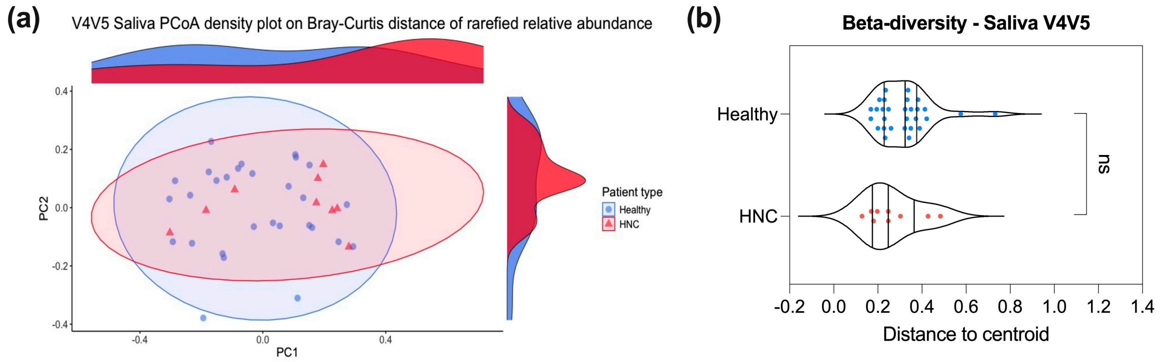

Figure S13. Comparison of V4V5 saliva beta-diversity between HNC, premalignant and healthy patient at the genus level. Based on conventional analysis method, raw abundance counts were rarefied and converted to relative abundance for (a) V4V5 saliva PCoA density plot on Bray-Curtis distance. Beta-diversity for each sample was calculated as distance to centroid for each patient type for (b) V4V5 saliva. Kruskal-Wallis test with Dunn's multiple comparison or Mann-Whitney test were performed to test for differences in beta-diversity. ****$p < 0.0001$, ***$p < 0.001$, **$p < 0.01$, *$p < 0.05$, ns – not significant.

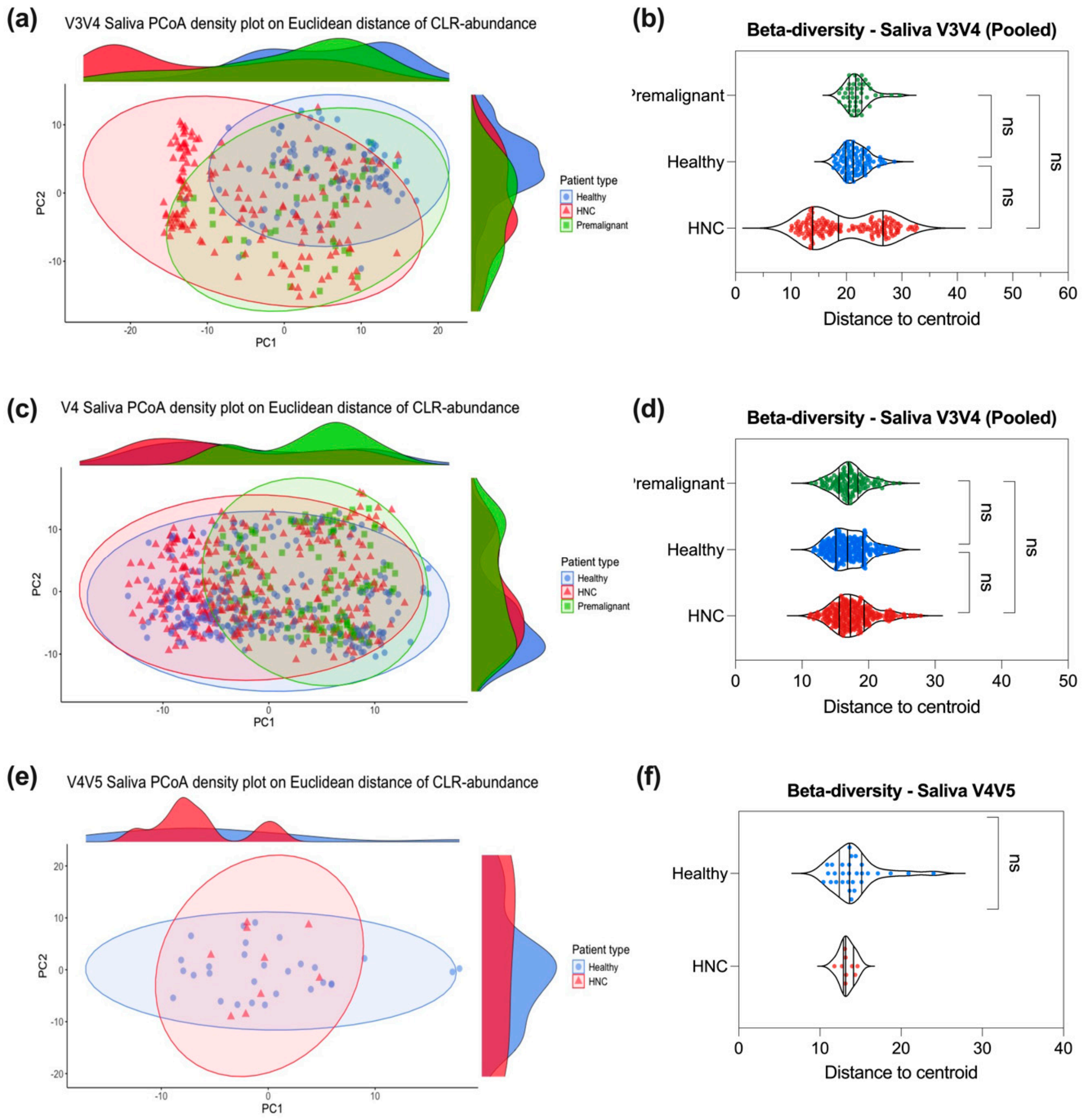

Figure S14. Comparison of saliva beta-diversity between HNC, premalignant and healthy patient at the genus level based on CLR-abundance. Based on CoDA method, raw abundance counts were converted to CLR-abundance (offset = 0.5), and CLR-abundance were used for (a) V3V4, (c) V4, and (e) V4V5 saliva PCoA density plot on Euclidean distance. Beta-diversity for each sample was calculated as distance to centroid for each patient type for (b) V3V4, (d) V4, and (f) V4V5 saliva. Kruskal-Wallis test with Dunn's multiple comparison or Mann-Whitney test were performed to test for differences in beta-diversity. ****$p < 0.0001$, ***$p < 0.001$, **$p < 0.01$, *$p < 0.05$, ns – not significant.

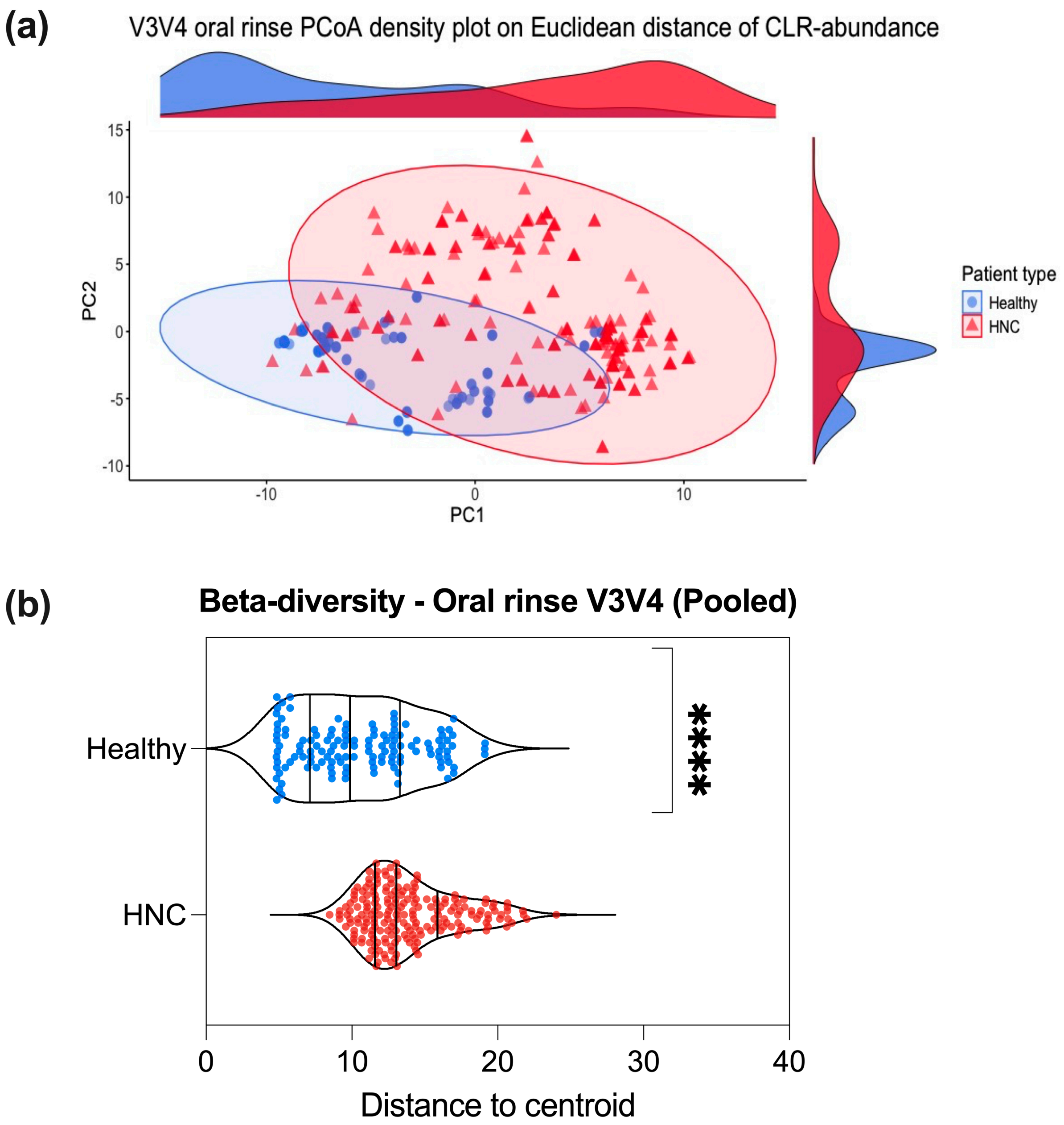

Figure S15. Comparison of oral rinse beta-diversity between HNC and healthy patient at the genus level based on CLR-abundance. Based on CoDA method, raw abundance counts were converted to CLR-abundance (offset = 0.5), and CLR-abundance were used for (a) V3V4 oral rinse PCoA density plot on Euclidean distance. Beta-diversity for each sample was calculated as distance to centroid for each patient type for (b) V3V4 oral rinse. Mann-Whitney test was performed to test for differences in beta-diversity. ****$p < 0.0001$, ***$p < 0.001$, **$p < 0.01$, *$p < 0.05$, ns – not significant.

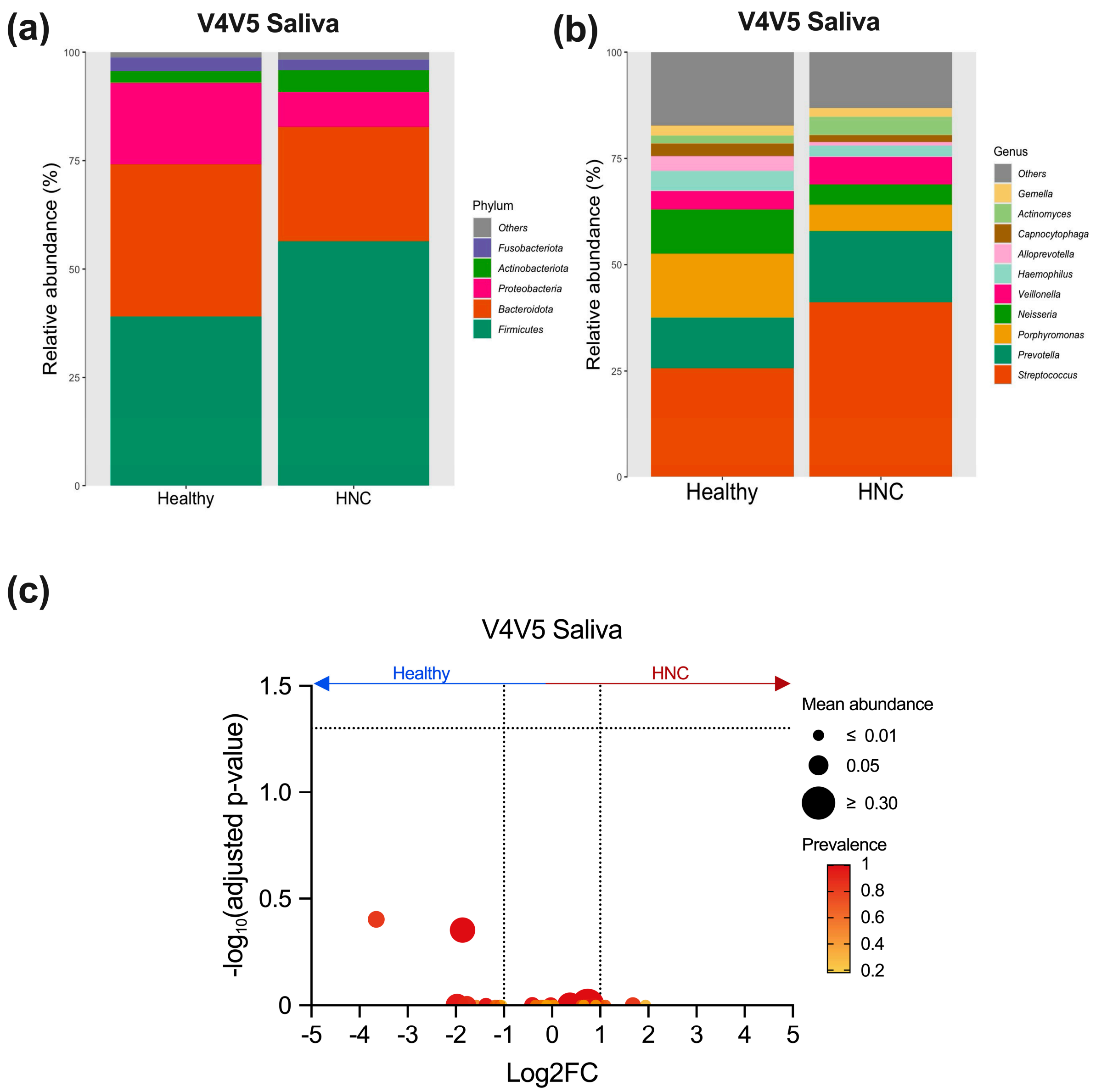

Figure S16. Microbial relative abundance and differential abundance analysis for V4V5 saliva samples. Mean relative abundance of V4V5 saliva samples at the (a) phylum and (b) genus level. Differential abundance analysis (LinDA) was performed to determine the differences between HNC to healthy saliva samples for (c) V4V5. P-values were adjusted for FDR using the Benjamini-Hochberg method.
